# A multimodal lifestyle intervention complemented with epigallocatechin gallate to prevent cognitive decline in *APOE*- ɛ4 carriers with Subjective Cognitive Decline: a randomized, double-blinded clinical trial (PENSA study)

**DOI:** 10.1016/j.tjpad.2025.100271

**Published:** 2025-07-15

**Authors:** Laura Forcano, Natalia Soldevila-Domenech, Anna Boronat, Gonzalo Sánchez-Benavides, Albert Puig-Pijoan, Thais Lorenzo, Ana Aldea-Perona, Marc Suárez-Calvet, Aida Cuenca-Royo, Juan Domingo Gispert, Maria Gomis-Gonzalez, Carolina Minguillón, Patrícia Diaz-Pellicer, Karine Fauria, Iris Piera, Klaus Langohr, Mara Dierssen, Nieves Pizarro, Esther Mur-Gimeno, Oriol Grau-Rivera, José Luis Molinuevo, Rafael de la Torre, the PENSA working group

**Affiliations:** a Integrative Pharmacology and Systems Neuroscience Research Group, Neuroscience Research Program, Hospital del Mar Research Institute, Barcelona, Spain; b CIBER de Fisiopatología de la Obesidad y Nutrición, Instituto de Salud Carlos III, Madrid, Spain; c Barcelonaβeta Brain Research Center (BBRC), Pasqual Maragall Foundation, Barcelona, Spain; d Department of Medicine and Life Sciences, Universitat Pompeu Fabra, Barcelona, Spain; e Clinical and Risk Factors for Neurodegenerative Diseases Research Group, Neuroscience Research Program, Hospital del Mar Research Institute, Barcelona, Spain; f CIBER de Fragilidad y Envejecimiento Saludable (CIBER-FES), Instituto de Salud Carlos III, Madrid, Spain; g Cognition and Behavior Unit, Department of Neurology, Hospital Del Mar, Barcelona, Spain; h Medicine Department, Universitat Autònoma de Barcelona, Barcelona, Spain; i ERA-Net on Cardiovascular Diseases (ERA-CVD) Consortium, Barcelona, Spain; j Neuroimaging of neurodegenerative disease and healthy aging Research Group, Neuroscience Research Program, Hospital del Mar Research Institute, Barcelona, Spain; k Clinical Research Unit, Neuroscience Research Program, Hospital del Mar Research Institute, Barcelona, Spain; l CIBER en Bioingeniería, Biomateriales y Nanomedicina, Instituto de Salud Carlos III, Madrid, Spain; m Centro Nacional de Investigaciones Cardiovasculares (CNIC), Madrid, Spain; n Department of Statistics and Operations Research, Universitat Politècnica de Catalunya BARCELONATECH, Barcelona, Spain; o Centre for Genomic Regulation (CRG), The Barcelona Institute of Science and Technology, Barcelona, Spain; p CIBER de Enfermedades Raras (CIBERER), Madrid, Spain; q Attention to Chronicity and Innovation in Health (GRACIS), Escola Superior de Ciències de la Salut Tecnocampus, Universitat Pompeu Fabra, Mataró, Spain; r Experimental Medicine, H. Lundbeck A/S, København, Denmark

**Keywords:** Multimodal lifestyle intervention, Prevention, APOE-ɛ4, Epigallocatechin-3-gallate, Subjective Cognitive Decline, Randomized Controlled Trial

## Abstract

**Background:**

The potential of dietary compounds to enhance the effects of multimodal lifestyle interventions (MLIs) on cognition in individuals at high risk of cognitive impairment remains unclear.

**Objectives:**

To assess whether the addition of a green tea extract enriched with epigallocatechin-3-gallate (EGCG) enhances the effects of an MLI.

**Design:**

Double-blind, randomized, two-arm, and placebo-controlled trial. Exploratory comparisons were made with a non-randomized group (NRG) receiving healthy lifestyle recommendations. **Setting:** Population-based study conducted in Barcelona, Spain

**Participants:**

*APOE*-ɛ4 carriers aged 60-80 with subjective cognitive decline

**Intervention:**

A 12-month intensive MLI including dietary counseling, guided physical activity, and cognitive stimulation, combined with EGCG (5-6 mg/kg) or placebo, followed by a 3-month washout.

**Measurements:**

Primary endpoint was change in the modified Preclinical Alzheimer Cognitive Composite (PACC-exe) score.

**Results:**

129 participants (65.1% 84 women, aged 66.7±5.5 years) were enrolled (52 MLI+EGCG, 52 MLI+placebo and 25 NRG), with126 (97.7%) included in the modified intention-to-treat analysis. After 12 months, no statistically significant difference was observed between MLI+EGCG and MLI+placebo in the PACC-exe (adjusted mean difference [AMD]: 0.12; 95%CI: -0.01, 0.24; p=0.061). However, participants in the MLI+EGCG group were 2.6 times more likely to show a reliable cognitive improvement. In exploratory analyses following a 3-month washout, the MLI+EGCG group showed significant cognitive benefits compared to the MLI+placebo (AMD: 0.19; 95%CI: 0.06, 0.32; p=0.005). Exploratory comparisons with the NRG also suggested greater gains in cognition and dementia risk reduction in both MLI groups, particularly with EGCG.

**Conclusions:**

While the primary outcome was not met, this proof-of-concept trial suggests that combining MLIs with EGCG warrants further investigation in larger, confirmatory studies.

## Highlights PENSA Study

• We explored the effects of MI combined with EGCG in *APOE*-ɛ4 carriers with SCD.
• Cognitive benefits from EGCG combined with MI persist after 3 months of treatment discontinuation.
• Combining lifestyle MI and EGCG shows promise for reducing dementia risk.
• EGCG may reduce dementia risk via mechanisms beyond amyloid and tau pathways.

**TRIAL REGISTRATION:** ClinicalTrials.gov Identifier NCT03978052

## Introduction

Alzheimer’s disease (AD), the leading cause of dementia, is a major healthcare and societal challenge with a long preclinical phase and no curative treatment at present (1). To address this challenge, AD prevention has become the main goal, considering the long period preceding the onset of objectively measurable deficits in cognition and functionality (1). About one in four individuals over 60 years old experience subjective cognitive decline (SCD), which may represent the first clinical symptom of AD (2). Although not all individuals with SCD progress mild cognitive impairment (MCI) or dementia, they are currently recognized as an especially well-suited target population for early intervention trials in AD (2). This is particularly relevant for those who also carry the major genetic risk factor for sporadic AD, the ɛ4 allele of the apolipoprotein E gene (*APOE*-ɛ4). Indeed, the *APOE*-ɛ4 allele increases the likelihood of preclinical AD pathology in individuals with SCD (2). Focusing on this high-risk population in prevention trials is particularly relevant, as it aligns with the concept of precision prevention, which aims to tailor interventions to those most likely to benefit. However, there has been no clinical trial conducted exclusively on *APOE*-ɛ4 carriers with SCD to prevent cognitive decline.

The rationale for AD prevention is reinforced by the identification of various modifiable risk factors, including vascular and metabolic impairments, psychosocial aspects, and lifestyle behaviors (3,4). While it is unclear whether these factors relate to AD pathology or overall dementia, they are promising targets for prevention. In this framework, the landmark Finnish Geriatric Intervention Study to Prevent Cognitive Impairment and Disability (FINGER) clinical trial showed that a lifestyle-based multimodal intervention (MLI) targeting several risk factors simultaneously (diet, physical activity, and cognitive training) improved cognition in people at increased risk of dementia (5). Later, the FINGER model has been adapted to diverse populations and is currently being tested in more than 60 countries under the World-Wide FINGERS network of clinical trials (6). However, to date only results from a limited number of WW-FINGER and aligned trials have been published ([7], [8], [9], [10], [11], [12], [13], [14], [15], [16], [17], [18]). While the FINGER trial remains as the sole large-scale MLI study to demonstrate significant benefits on the primary cognitive outcome,(5) some more recent trials -many of which are part of the WW-FINGER network- have also reported positive effects either on primary cognitive endpoints, or in other cognitive secondary or exploratory outcomes.

Among the published trials, the J-MINT PRIME Tamba (13), COMBAT (14), and StayFitLonger (15) studies reported significant benefits on primary cognitive endpoints in older adults at elevated risk. In contrast, no such effects were found in the AgeWell.de (8), SMARRT (9), or J-MINT (12) trials. Other studies such as GOIZ ZAINDU (7), MIND-ADmini (11), and the Korean study by Moon et al. (10) did not include cognition as a primary endpoint.

Exploratory or secondary analyses indicated cognitive gains in some subgroups or domains: executive function (GOIZ ZAINDU), global cognition in per-protocol or high adherence participants (J-MINT, SMARRT), APOE-ε4 carriers (MIND-ADmini), and improved memory and attention in a remotely coached MLI intervention (16). Similarly, post-hoc analyses of two large-scale trials (FINGER and MAPT) have shown that individuals at increased risk of future cognitive decline—such as APOE-ε4 carriers or those with positive amyloid PET scans—tend to derive greater cognitive benefits from MLI compared to non-carriers. This suggests that targeting high-risk individuals, particularly in the early, non-symptomatic stages of Alzheimer’s disease, instead of unselected populations might be the most effective approach for multimodal prevention trials (17). In addition, general lifestyle advice may not suffice; instead, detailed guidance or training are necessary to impact cognition (17).

A new generation of precision prevention studies -FINGER 2.0 trials- aims to maximize the benefits of lifestyle-based MLIs by combining them with pharmaceuticals/nutraceuticals with pro-cognitive effects (11,18). Epigallocatechin-3-gallate (EGCG), a flavonoid in green tea, has shown promise as a neuroprotective agent targeting several mechanisms underlying neurodegeneration in AD (19). EGCG acts as an antioxidant, regulates inflammatory processes, triggers neuronal survival pathways and influence metabolic and vascular pathways that contribute to cognitive decline (19). Preclinical studies suggest that EGCG can reduce amyloid-beta accumulation, inhibit tau phosphorylation, and improve synaptic plasticity. In clinical studies, such as those involving young adults with Down syndrome, EGCG combined with cognitive training resulted in a mild increase in cognitive performance and adaptive functionality, sustained after treatment discontinuation (20). Furthermore, its potential impact on insulin resistance and vascular function highlights its relevance for addressing modifiable risk factors associated with AD beyond amyloid and tau pathways ([21], [22], [23]). These findings, together with its good safety profile (24), could make EGCG a potential pro-cognitive therapeutic compound for preventing neurodegenerative conditions such as AD. In line with the FINGER 2.0 model, the combination of an MLI with EGCG could represent a therapeutic approach for AD prevention.

Given previous evidence of the efficacy of MLI (5,9,10,[14], [15], [16],[25], [26], [27]), we conducted a proof-of-concept trial to investigate the efficacy of combining EGCG with an intensive MLI for 12 months to prevent cognitive decline in *APOE*-ɛ4 carriers with SCD. The primary objective of the PENSA study was to evaluate the efficacy of an intensive MLI supplemented with EGCG compared to the same MLI supplemented with placebo in this at-risk population, focusing on cognitive performance measured by the modified Preclinical Alzheimer Cognitive Composite (PACC-exe). Additionally, an exploratory analysis was conducted to contextualize the cognitive trajectories of the MLI groups by comparing them with a non-randomized reference group (NRG) that received general lifestyle recommendations, providing insights into the expected natural cognitive trajectories. This study contributes to the emerging field of precision prevention by focusing on a well-defined at-risk population and exploring the synergistic effects of combining lifestyle interventions with bioactive nutraceuticals.

## Methods

The PENSA Study was a two-arm randomized, double-blind, placebo-controlled clinical trial involving an MLI with EGCG or Placebo (ClinicalTrials.gov Identifier NCT03978052). The trial assessed the efficacy of a 12-month MLI with EGCG (MLI+EGCG) in comparison with MLI with placebo EGCG (MLI+placebo). A NRG receiving general lifestyle recommendations was also included to support exploratory comparisons. An additional 3-month follow-up, once intervention was discontinued, allowed to evaluate to which extent potential beneficial effects were sustained overtime time. The study was performed in Barcelona (Spain), at the Hospital del Mar Research Institute (HMRI) in collaboration with the Barcelonaβeta Brain Research Center (BBRC)(28). The institutional review board approved the protocol (CEIm-Hospital del Mar Barcelona, IMLIM/PENSA) and has been registered as a clinical trial (ClinicalTrials.gov Identifier NCT03978052). All participants provided written informed consent. This study followed the Consolidated Standards of Reporting Trials (CONSORT) reporting guidelines.

### Study participants

Participants were primarily recruited from the general population through a web-based platform, where they registered and completed an initial screening questionnaire. The original study design intended to include three randomized groups: MLI+EGCG, MLI+placebo, and a control group receiving general lifestyle recommendations. However, the recruitment process, which commenced shortly before the COVID-19 pandemic, encountered significant challenges due to government-imposed restrictions and public health concerns. Despite adapting participant screening procedures, including home-based screening visits, recruitment for all three randomized arms became increasingly difficult. Given the need to adhere to the planned recruitment timeline, a strategic decision was made to prioritize the randomization of participants into the two active intervention groups (MLI+EGCG and MLI+placebo), as the primary objective of the study was to assess the added value of EGCG to a well-established multimodal intervention. Thus, individuals who expressed interest in participating but preferred not to engage in all aspects of the intensive intervention—primarily due to concerns regarding group-based activities, mobility limitations, or potential relocation—were included in a non-randomized reference group (NRG). This group received general lifestyle recommendations and served as an exploratory reference to contextualize the cognitive trajectories observed in the intervention groups only in secondary exploratory analyses.

Pre-selection criteria targeted adults aged 60-80 with self-perceived cognitive decline assessed through an ad-hoc online questionnaire specifically designed to evaluate SCD. Candidates meeting these initial criteria were invited to a pre-screening visit, which included oral fluid collection for *APOE* genotyping. *APOE-ɛ4* carriers were then invited to an *APOE* risk communication visit (28), conducted according to a standardized protocol, and subsequently invited to participate in the study. Additional eligibility assessments were then carried out. These included fulfilling at least three of the following SCD *plus* features (29) from a second SCD questionnaire administered by a neurologist or neuropsychologist (Supplementary material): memory-centered decline, persistent decline within the last five years, SCD corroborated by an informant, concern about cognitive decline, and perception of lower performance in comparison with same age group. Moreover, their cognitive performance (assessed using the same neuropsychological battery later used for outcome evaluation) should be within normal ranges (all psychometric scores above -1.5 standard deviations (SD), according to age- and education-adjusted norms), their body mass index (BMI) between ≥18.5 and <35 kg/m^2^, and they had to present no significant structural abnormalities, such as brain infarcts, tumours, aneurysms, cysts, or malformations, on neuroimaging. Participants with large confluent white matter hyperintensities (WMH), as denoted by a Fazekas score of 3, were excluded as this degree of WMH is always considered pathological, regardless of age (30). No financial compensation was provided for participation.

Although the web-based recruitment strategy allowed for wide geographical access, it may have inadvertently limited participation from individuals with lower socioeconomic status or lower digital literacy. Consequently, the study population was predominantly composed of highly educated individuals, with the majority identifying as White. This underrepresentation of racial, ethnic, and socioeconomic diversity may limit the generalizability of the findings to broader populations.

### Randomization and masking

Participants who were willing to participate in the MLI were randomized to EGCG or placebo in a 1:1 ratio sequentially in the order in which they were enrolled. Randomization sequences were generated by an independent statistician (KL) separately for female and male participants in blocks of four individuals (two of each sex randomly allocated to each group) and uploaded into the electronic Case Report Form (eCRF), which automatically assigned a treatment group to each participant upon inclusion. Exclusive access to treatment allocation was granted to the Pharmacy Department of Hospital del Mar, responsible for provisioning identical presentations of active and placebo treatments. Outcome evaluators were not involved in intervention activities and were blinded to the study arms. Participants were instructed not to discuss the intervention with outcome evaluators. The double blind was maintained until the study eCRF was closed. Statistical analyses were done without unblinding the MLI groups.

### Sample collection

The intervention lasted 12 months, with a follow-up visit at 15 months to assess the stability/reversibility of changes, including neuropsychological evaluation and self-reported questionnaires. Participants met the study neurologist at the screening visit and 6, 12, and 15 months for a medical history and physical exams, and the study nurse at baseline, 6, and 12 months for blood sample collection, biochemical analysis, blood pressure, anthropometrics, body composition (by bioelectrical impedance analysis), and electrocardiogram evaluation. Magnetic resonance imaging (MRI), blood-based AD biomarkers, homocysteine, and olfactory function assessments were performed at baseline and after 12 months.

### Intervention

MLI participants were grouped into 10-15 people sub-groups, received an induction session, and a comprehensive ‘study guidebook’ containing detailed information on study procedures and visits and on healthy lifestyle behaviors. During a run-in period of 15-30 days baseline information about their diet, physical activity, and sleep, to personalize the MLI was collected. All participants had a chance to contact the study nurse by telephone or e-mail when needed. NRG participants met the same inclusion criteria as participants in the MLI, including the fact that they were carriers of the *APOE*-ɛ4 allele and received oral and written healthy lifestyle recommendations.

Participants received either EGCG or placebo in oral capsules, taken daily over a 12-month period. Each active capsule contained 225.5 mg of decaffeinated green tea extract, providing100 mg of EGCG (min 94% purity of EGCG). The placebo capsules were identical in appearance and packaging, and contained 345 mg of maltodextrin. Both were supplied by Laboratoires Grand Fontaine (FontUp capsules, FontActiv®). The total daily dose of EGCG was determined based on participants’ body weight: those weighing 50 kg or less received 300 mg/day (3 capsules), while those weighing more than 50 kg received 500 mg/day (5 capsules). This dosing remained within the maximum daily intake of 600 mg EGCG/day recommended by health authorities (31). Treatment compliance was assessed by analyzing EGCG in plasma samples at 6 and 12 months using liquid chromatography coupled with tandem mass spectrometry (LC-MS/MS).

The intensive and personalized MLI included diet, physical activity, and mental health (cognitive training, psychoeducation, and social stimulation). For detailed descriptions of each component, please refer to the published protocol (28) or see Supplementary material. The nutritional intervention was based on the Mediterranean diet (MedDiet) and supervised by a nutritionist (two individual sessions during the run-in period and nine during the intervention period). Participants received monthly personalized reports to reinforce MedDiet adherence based on the data collected through daily ecological momentary assessments (EMAs). They also benefited from supermarket discounts on selected MedDiet products. The physical activity intervention included 60-minute group-based gymnasium classes guided by a physiotherapist or a certified sports coach (one session/week during the first semester and two sessions/week during the second semester). Participants wore activity trackers to self-monitor and increase steps/day and active minutes. Cognitive training involve 30-minute sessions trice/week delivered through NeuronUP© telematic platform (https://www.neuronup.us/) (32). The psychoeducation program consisted of ten 90-minute group sessions led by experienced psychologists. The social stimulation intervention included ten 90-120 minutes sessions led by professionals based on the session topic. Participant satisfaction with the intervention was assessed at month 6 and after every psychoeducational and social stimulation session, focusing on usability/ usefulness, adherence, and overall satisfaction. Feedback was used to make minor adjustments to enhance engagement.

### Outcomes

The primary efficacy endpoint was the change in cognitive performance at 12 months measured by the modified Preclinical Alzheimer Cognitive Composite (33) including additional tests of executive functions (PACC-exe) (28). This composite included six tests: the Free and Cued Selective Reminding Test–Total Recall, Logical Memory Delayed Recall, Coding subtest from WAIS-IV, Stroop interference score, Five Digit Test, and the Montreal Cognitive Assessment (MoCA). Executive function measures were incorporated to improve sensitivity to early Alzheimer-related decline, as this domain is increasingly recognized as vulnerable in preclinical stages of the disease (34) (See Supplementary material for further details). Higher scores on the PACC-exe indicate better cognitive performance.

Secondary endpoints included evaluating treatment safety and tolerability through incidence, nature, severity, and causality of adverse events (AEs) and serious adverse events (SAEs). AEs and SAEs were documented using the Medical Dictionary for Regulatory Activities (MedDRA) version 24.0. Secondary endpoints also included changes in structural magnetic resonance imaging (MRI), assessing hippocampal and lateral ventricles volumes, cortical thickness, and White Matter Hyperintensities (WMH) (Supplementary material).

Exploratory efficacy endpoints included: sustainability of cognitive changes at 15 months; mean differences between groups in the Montreal Cognitive Assessment (MoCA) total score, memory and executive functioning composites evaluated with the whole PENSA study neuropsychological battery; and cognitive decline and cognitive improvement, defined according to reliable change indexes from standardized regression-based formulas (RCI_SRB_ ± 1.645) (35).

Additional exploratory outcomes included blood-based biomarkers of AD (Aβ42/40, p-tau181, GFAP, and NfL), plasma homocysteine (i.e., a biomarker of EGCG activity via the Dual Specificity Tyrosine-Phosphorylation-Regulated Kinase 1A —Dyrk1A and its interaction with One-carbon (1C) metabolism) (36), olfactory functioning, dementia risk scores (Cardiovascular Risk Factors, Aging, and Dementia —CAIDE index; and the Lifestyle for Brain Health—LIBRA index), cardiovascular and glycemic risk factors, changes in lifestyle behaviors and physical fitness, and everyday life functioning assessed with questionnaires for the following domains: adaptive behavior, quality of life, quality of sleep, and psychological distress. Adherence to the MLI was based on compliance with EGCG, participation in offered activities (mean proportion of the completed sessions), and compliance with eHealth devices. A detailed description of all study variables is included in Supplementary material.

For comparison purposes, an exploratory analysis comparing cognitive performance and dementia risk trajectories in the NRG with those observed in the randomized intervention groups (MLI+EGCG and MLI+placebo) has been conducted. The analysis focuses only on the primary cognitive outcome (ADCS-PACC-exe), and the dementia risk profile measured by the LIBRA score.

### Statistical analysis

Baseline characteristics of all included study participants are presented separately for each study group (MLI + EGCG, MLI + Placebo, and Non-Randomized Group [NRG]) as means and standard deviations for quantitative variables, and as absolute and relative frequencies for qualitative variables. In addition, absolute and relative frequencies of adverse effects are reported for each study group.

For the principal study objective —comparing the two randomized MLI groups after 12 months of intervention with respect to primary and exploratory cognitive endpoints— ANCOVA models were used, with change from baseline as the response variable and both treatment group and baseline score as predictors. The corresponding effect size was expressed as the baseline score-adjusted mean difference (AMD) between the two treatments. Additionally, to quantify differences in change from baseline on a standardized scale, Hedges’ g was calculated. Average changes within each study group were also computed, along with corresponding 95% confidence intervals (95% CI). The same analyses were repeated for changes observed after an additional 3-month washout period.

For the additional exploratory analysis —comparing both MLI groups with the NRG— changes from baseline were analyzed after the intervention period and following the additional 3-month washout. ANCOVA models were applied, further adjusted for baseline differences in sex, years of education, and alcohol consumption between the MLI groups and the NRG. For post-hoc comparisons, Dunnett’s method was used to control multiple testing.

Treatment effects for each outcome and for both intervention and washout period were assessed in the modified intention-to-treat (mITT) population, which included all participants with the corresponding post-baseline assessment.

Additionally, binary logistic regression models were used to evaluate differences between MLI treatments (i.e., MLI+EGCG vs. MLI+placebo) in the risk of reliable cognitive decline and improvement at 12 months, using the MLI+placebo group as the reference.

Statistical significance was set at *p* < 0.05. All analyses were conducted using R software, version 4.4.2 (The R Foundation for Statistical Computing, Vienna, Austria).

## Results

### Participant recruitment, allocation, and retention

**Figure 1:**
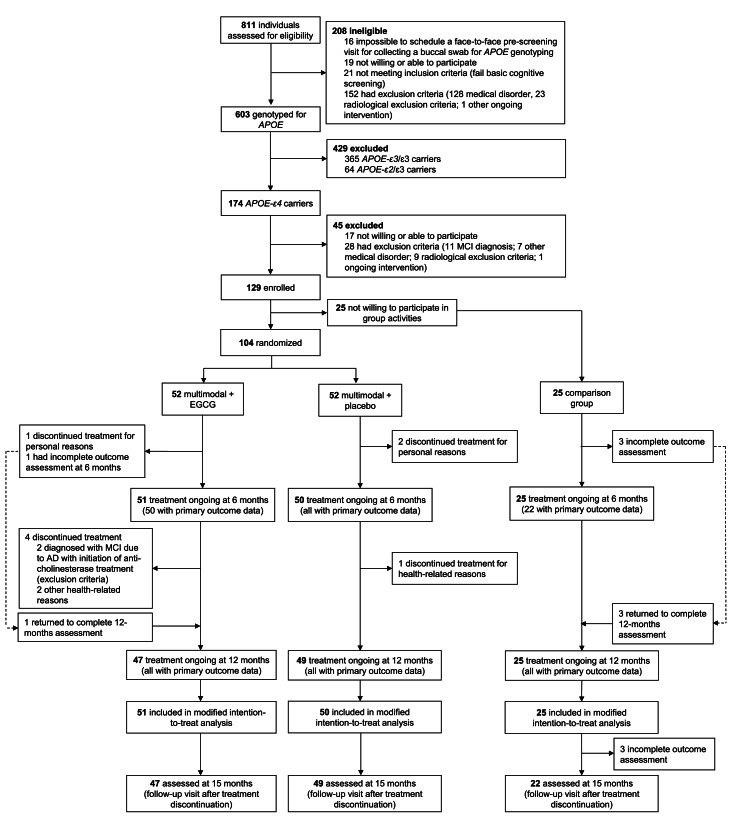
Trial profil**e** AD= Alzheimer’s disease. APOE= Apolipoprotein E genotype. EGCG= epigallocatechin-3-gallate. MCI= mild cognitive impairment.

Between November 5, 2019, and January 26, 2022, 811 individuals meeting pre-selection inclusion criteria were screened from a pool of 1329 individuals registered on the website of the project. Of these, 423 did not meet the web-based pre-selection criteria and 95 could not be contacted after several attempts. Out of the initial 603 participants who were pre-screened and genotyped for *APOE*, 174 were identified as *APOE*-ɛ4 carriers, with 129 finally included (Figure 1). From these, 104 were randomly assigned to an MLI+EGCG (n=52) or a MLI+placebo (n=52), and 25 did not want to participate in group activities and were assigned to the NRG. The MLI was completed in March 2023. Eight participants withdrew from the study. The mITT analysis included 126 participants (97.7% of all enrolled participants), 51 from the MLI+EGCG, 50 from the MLI+placebo and 25 from the NRG. The follow-up visit at 15 months was performed in the mITT population, although this visit was not possible to schedule for three participants from the NRG. Two participants from the MLI+EGCG group (3.8 % subjects of this arm) were diagnosed with MCI during the intervention, three further subjects from the NRG (12.0% subjects of this arm) were diagnosed in the follow-up period, at 15 months (MCI diagnostic protocol is detailed in the Supplementary material).

### Baseline Demographics

Baseline characteristics are displayed in Table 1. Participants were predominantly female, in their late 60s, with high education, and the majority reported family history of dementia. Almost all participants self-identified as White. The MLI+EGCG group had a higher prevalence of type 2 diabetes, a history of depression, and a lower cerebrovascular disease burden (WMH). Groups showed similar SCD characteristics (Supplement 1, Appendix 2, Table 1).

**Table 1:** Baseline characteristics of included participants.

	MLI+EGCG (n=52)	MLI+placebo (n=52)	NRG (n=25)
*Sociodemographic characteristics*			
Sex*			
Female	36 (69.2)	33 (63.5)	15 (60.0)
Male	16 (30.8)	19 (36.5)	10 (40.0)
Age at the baseline visit (years)	67.1 (4.7)	67.6 (4.6)	66.7 (5.5)
*APOE* genotype			
ɛ2/ɛ4	4 (7.7)	4 (7.7)	1 (4.0)
ɛ3/ɛ4	44 (84.6)	47 (90.4)	20 (80.0)
ɛ4/ɛ4	4 (7.7)	1 (1.9)	4 (16.0)
Education (years)	15.0 (3.7)	14.3 (3.7)	16.2 (2.8)
Retired	42 (80.8)	47 (90.4)	15 (60.0)
Living alone	15 (28.8)	13 (25.0)	3 (12.0)
Family history of dementia	38 (73.1)	38 (73.1)	17 (68.0)
Family history of AD	26 (50.0)	32 (61.5)	12 (48.0)
*Dementia risk scores*			
CAIDE	7.5 (2.0)	7.5 (2.0)	7.7 (1.8)
LIBRA index	-0.4 (2.4)	-0.5 (2.3)	-0.1 (2.5)
*Lifestyle factors*			
MedDiet adherence score	7.8 (1.8)	8.0 (1.9)	8.0 (2.0)
High MedDiet adherence	10 (19.2)	12 (23.1)	6 (24.0)
Physical activity (METs-min/week)	3512.2 (2400.5)	3198.9 (1794.0)	3127.9 (2464.1)
Physical activity status			
Sedentary	1 (1.9)	1 (1.9)	1 (4.0)
Moderately active	6 (11.5)	8 (15.4)	5 (20.0)
Active	15 (28.8)	10 (19.2)	6 (24.0)
Very active	30 (57.7)	33 (63.5)	13 (52.0)
Alcohol consumption (g/week)	66.6 (98.7)	60.1 (72.0)	120.9 (125.8)
Cognitive reserve	16.8 (3.7)	16.7 (4.0)	17.4 (3.2)
Activation (PAM-13 level)			
Very Low	1 (1.9)	1 (1.9)	2 (8.0)
Low	9 (17.3)	6 (11.5)	3 (12.0)
Medium	26 (50.0)	29 (55.8)	12 (48.0)
High	16 (30.8)	16 (30.8)	8 (32.0)
Current smoker	4 (7.7)	4 (7.7)	2 (8.0)
High risk of sleep apnea	8 (15.4)	9 (17.6)^†^	7 (28.0)
*Self-reported medical disorders*			
Diabetes	10 (19.2)	1 (1.9)	4 (16.0)
History of Depression	20 (38.5)	12 (23.1)	3 (12.0)
Hypertension	16 (30.8)	20 (38.5)	11 (45.8)^†^
History of CVD	17 (32.7)	24 (46.1)	11 (44.0)
History of stroke	0 (0.0)	0 (0.0)	1 (4.0)
*Vascular risk factors*			
Metabolic Syndrome	17 (32.7)	16 (30.8)	8 (36.4)^†^
BMLI (kg/m^2^)	25.9 (4.6)	26.2 (3.9)	24.4 (4.6)
Systolic BP (mmHg)	131.1 (16.2)	132.5 (11.9)	137.7 (19.2)
Diastolic BP (mmHg)	69.9 (12.9)	72.7 (11.1)	73.4 (10.3)
Total cholesterol (mg/dL)	205.5 (32.7)	205.2 (35.6)	217.5 (34.6)
Fasting plasma glucose (mg/dL)	100.3 (24.2)	96.0 (12.5)	100.6 (18.6)
HbA1c (%)	5.69 (0.63)	5.52 (0.40)	5.54 (0.33)
*Cognition, mean (SD)*			
PACC-exe (Z score)	-0.03 (0.66)	0.01 (0.59)	0.05 (0.81)
Memory composite (Z score)	-0.05 (0.83)	0.07 (0.72)	-0.06 (0.90)
Executive functioning composite (Z score)	0.06 (0.65)	-0.10 (0.69)	0.09 (0.77)
MMSE total score	28.9 (1.4)	29.0 (1.1)	28.6 (1.2)
MoCA total score	26.5 (2.6)	26.7 (2.3)	27.0 (2.5)
*AD biomarkers, median [IQR]*			
Plasma Aβ40 (pg/mL)	111.5 [101.8, 122.5]	115.5 [104.5, 130.0]	117.0 [101.0, 129.0]
Plasma Aβ42 (pg/mL)	5.6 [4.7, 6.6]	5.5 [4.7, 6.5]	5.7 [4.8, 6.7]
Plasma Aβ42/40 ratio	0.050 [0.044, 0.057]	0.047 [0.044, 0.053]	0.052 [0.042, 0.055]
Plasma p-tau181 (pg/mL)	24.6 [19.6, 31.6]	23.9 [19.8, 29.8]	27.1 [22.1, 37.8]
Plasma NfL (pg/mL)	20.3 [16.8, 28.1]	20.3 [17.2, 23.5]	22.9 [17.1, 30.4]
Plasma GFAP (pg/mL)	96.2 [77.1, 156.0]	115.0 [79.0, 160.5]	121.0 [90.1, 153.0]
*Brain structure (MRI), median [IQR]*			
Hippocampal volume / TIV (%)	0.428 [0.409, 0.445]	0.427 [0.412, 0.460]	0.424 [0.397, 0.440]
Ventricular volume / TIV (%)	1.638 [1.219, 2.114]	1.719 [1.243, 2.055]	1.942 [1.424, 2.252]
Cortical thickness AD signature (mm)	2.428 [2.378, 2.485]	2.419 [2.366, 2.489]	2.419 [2.397, 2.462]
WMH / TIV (%)	0.152 [0.099, 0.244]	0.220 [0.120, 0.424]	0.196 [0.116, 0.316]

Values are n (%) for categorical variables and mean (SD) for continuous variables (except AD biomarkers that are median [IQR]). Results are unweighted. The sum of percentages does not always add up exactly to 100% because of rounding.

*Self-identified sex. ^†^n=1 participant had missing value.

AD= Alzheimer’s disease. APOE= apolipoprotein E. A = amyloid beta. BP= blood pressure. CAIDE= Cardiovascular Risk Factors, Aging, and Dementia index. NRG= non-randomized comparison group. CVD= cardiovascular disease. EGCG= epigallocatechin-3-gallate. GFAP= Glial fibrillary acidic protein. HbA1c= glycosylated hemoglobin. LIBRA= Lifestyle for Brain Health. MedDiet= Mediterranean diet. METs= metabolic equivalent tasks. MLI=multimodal intervention. MMSE= Mini-Mental State Exam. MoCA= Montreal Cognitive Assessment. NfL= neurofilament light. PACC-exe= modified preclinical Alzheimer’s Cognitive Composite including additional tests of executive functioning. PAM-13= Patient Activation Measure. p-tau-181= phosphorylated tau 181. TIV= total intracranial volume. WMH= white matter hyperintensities.

### Cognitive Outcomes

**Figure 2:**
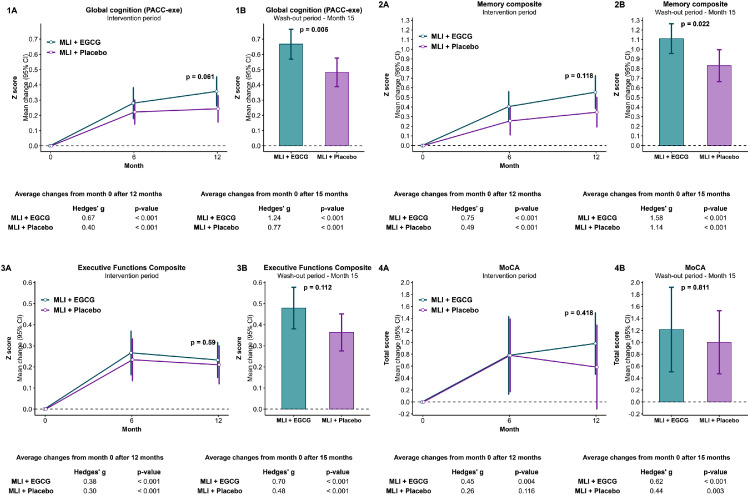
Changes in global cognition, memory, executive functions, and MoCA scores during the intervention and follow-up periods in participants receiving MLI + EGCG versus MLI + Placebo. Change in the primary cognitive endpoint during the 12 months intervention (1A) and after a wash-out period of three months (1B); change in the memory composite during the 12 months intervention (2A) and after a 3-months wash-out period (2B); change in the executive functioning composite during the 12 months intervention (3A) and after a 3-months wash-out period (3B); and change in the MoCA total score during the intervention (4A) and after 3-months treatment discontinuation (4B). *EGCG= epigallocatechin-3-gallate. MLI=multimodal intervention. MoCA= Montreal cognitive assessment. PACC-exe= preclinical Alzheimer cognitive composite plus additional tests of executive functions*.

As shown in Table 2 and Figure 2, both MLI groups demonstrated significant improvements in cognitive performance from baseline. At 12 months, the estimated mean change in PACC-exe Z-score was 0.36 (95% CI: 0.26, 0.45) in the MLI+EGCG group and 0.24 (95% CI: 0.16, 0.33) in the MLI+placebo group. This represents a 50% greater improvement in the MLI+EGCG group, corresponding to a Hedge’s g effect size of 0.36. However, the adjusted mean difference (AMD) between the two groups was not statistically significant (AMD: 0.12; 95% CI: -0.01, 0.24; p = 0.061), indicating a null result for the primary cognitive outcome. Exploratory analyses of individual-level change patterns showed that participants in the MLI+EGCG group were 2.6 times more likely (95% CI: 1.1, 6.2) to exhibit a reliable cognitive improvement on the PACC-exe than those in the MLI+placebo group (Table 3). Additionally, the MLI+EGCG group showed non-statistical numerically greater improvement on the Boston Naming Test at 12 months (Hedges' g = 0.32; p = 0.063), a measure not included in the PACC-exe composite.

**Table 2:** Treatment effects on cognitive and health outcomes in the modified intention-to-treat population.

		MLI+EGCG (n=51)	MLI+placebo (n=50)	MLI+EGCG vs. MLI+placebo					
	Month	Mean	Mean Change (95%CI)	Mean	Mean Change (95%CI)	Adjusted mean difference* of changes from month 0 (95%CI)	p-value	Hedges’ g effect size	Favors MLI + EGCG (+)
Cognition									
PACC-exe (Z score)	0	-0.05		0.01					
	12	0.38	0.36 (0.26, 0.45)	0.25	0.24 (0.16, 0.33)	0.12 (-0.01, 0.24)	0.061	0.36	+
	15	0.69	0.67 (0.57, 0.76)	0.50	0.48 (0.39, 0.58)	0.19 (0.06, 0.32)	0.005	0.56	+
Memory composite (Z score)	0	-0.09		0.10					
	12	0.53	0.55 (0.38, 0.73)	0.45	0.35 (0.19, 0.50)	0.16 (-0.04, 0.37)	0.118	0.37	+
	15	1.09	1.11 (0.96, 1.26)	0.93	0.83 (0.66, 0.99)	0.24 (0.04, 0.44)	0.022	0.51	+
Executive functions composite (Z score)	0	0.04		-0.11					
	12	0.35	0.23 (0.15, 0.32)	0.12	0.21 (0.12, 0.30)	0.03 (-0.09, 0.16)	0.590	0.08	+
	15	0.60	0.48 (0.38, 0.58)	0.27	0.36 (0.28, 0.45)	0.11 (-0.03, 0.24)	0.112	0.36	+
MoCA total score	0	26.43		26.84					
	12	27.62	0.98 (0.46, 1.50)	27.42	0.58 (-0.12, 1.29)	0.29 (-0.420, 1.003)	0.418	0.18	+
	15	27.85	1.21 (0.50, 1.92)	27.86	1.00 (0.47, 1.53)	0.08 (-0.586, 0.747)	0.811	0.10	+
MMSE total score	0	28.92		29.0					
	12	29.19	0.11 (-0.27, 0.48)	29.2	0.16 (-0.26, 0.59)	-0.02 (-0.42, 0.38)	0.924	-0.04	
	15	28.89	-0.19 (-0.67, 0.29)	29.0	-0.06 (-0.51, 0.39)	-0.10 (-0.67, 0.47)	0.727	-0.08	
Semantic Fluency Test (Animals in 1 minute)	0	23.88		22.72					
	12	24.81	0.62 (-0.53, 1.76)	22.98	0.04 (-1.19, 1.27)	0.84 (-0.80, 2.47)	0.312	0.14	+
	15	25.55	1.36 (0.09, 2.63)	22.08	-0.92 (-2.26, 0.43)	2.52 (0.71, 4.32)	0.007	0.50	+
Boston Naming Test	0	13.80		13.84					
	12	14.34	0.40 (0.16, 0.65)	13.98	0.10 (-0.20, 0.40)	0.33 (-0.02, 0.67)	0.063	0.32	+
	15	14.55	0.62 (0.35, 0.88)	14.50	0.60 (0.34, 0.86)	0.04 (-0.23, 0.31)	0.790	0.01	+
Dementia risk scores, anthropometric factors, and cardiovascular risk factors									
LIBRA index	0	-0.45		-0.51					
	12	-1.96	-1.35 (-1.83, -0.87)	-1.67	-1.19 (-1.80, -0.59)	-0.20 (-0.89, 0.50)	0.579	-0.09	+
BMI (kg/m^2^)	0	25.84		26.26					
	12	25.22	-0.35 (-0.62, -0.08)	25.74	-0.56 (-1.04, -0.08)	0.14 (-0.38, 0.66)	0.603	0.16	
Muscular-to-fat mass ratio	0	2.47		2.43					
	12	2.69	0.17 (0.03, 0.31)	2.59	0.10 (-0.05, 0.26)	0.07 (-0.14, 0.27)	0.514	0.13	+
Visceral fat index	0	10.69		10.94					
	12	10.02	-0.68 (-1.15, -0.21)	10.74	-0.43 (-0.80, -0.07)	-0.31 (-0.85, 0.23)	0.253	-0.17	+
LDL-c/HDL-c ratio	0	2.20		2.27					
	12	2.12	-0.08 (-0.21, 0.05)	2.11	-0.15 (-0.26, -0.05)	0.06 (-0.09, 0.21)	0.446	0.17	
Triglycerides (mg/dL)	0	108.75		105.38					
	12	93.86	-14.88 (-23.73, -6.03)	92.82	-12.56 (-24.12, -1.00)	-1.04 (-13.15, 11.08)	0.866	-0.06	+
HOMA-IR index	0	2.91		2.23					
	12	2.14	-0.56 (-1.28, 0.16)	2.38	0.14 (-0.27, 0.55)	-0.46 (-1.09, 0.16)	0.145	-0.36	+

EGCG= epigallocatechin-3-gallate. MLI= multimodal intervention. MMSE= mini-mental state examination. MoCA= Montreal cognitive assessment. PACC-exe= preclinical Alzheimer’s cognitive composite plus additional measures of executive functions.

*Adjusted mean differences (AMD) are obtained from ANCOVA models for differences from baseline adjusted for baseline scores. In the case of positive changes from baseline, a positive value of the AMD indicates larger changes in the MLI+EGCG group. In the case of negative changes from baseline, a negative value of the AMD indicates larger absolute changes in the MLI+EGCG group.

Hedges’ g quantifies the difference in change from month 0 between the two groups on a standardized scale.

The results favor the MLI+EGCG group over MLI+placebo if the MLI+EGCG group shows greater positive changes.

**Table 3:** Risk of cognitive decline and cognitive improvement from baseline to 12 months between MLI groups in the modified intention-to-treat population.

		Cognitive decline (RCI_SRB_ ≤ -1.645)	Cognitive improvement (RCI_SRB_ ≥ 1.645)				
Cognitive domain	Group	n (%)	Odds ratio (95%CI)	p-value	n (%)	Odds ratio (95%CI)	p-value
Global cognition (PACC-exe)	MLI+placebo	22 (45.8%)	Reference category		13 (27.1%)	Reference category	
	MLI+EGCG	16 (34.0%)	0.6 (0.3, 1.4)	0.242	23 (47.9%)	2.6 (1.1, 6.2)	0.030
Memory composite	MLI+placebo	24 (50.0%)	Reference category		17 (35.4%)	Reference category	
	MLI+EGCG	15 (31.9%)	0.5 (0.2, 1.1)	0.075	21 (44.7%)	1.5 (0.6, 3.4)	0.357
Executive composite	MLI+placebo	19 (38.8%)	Reference category		17 (34.7%)	Reference category	
	MLI+EGCG	21 (44.7%)	1.3 (0.6, 2.9)	0.558	20 (42.5%)	1.4 (0.6, 3.2)	0.430

EGCG= epigallocatechin-3-gallate. MLI= multimodal intervention. RCISRB= reliable change index from standardized regression-based formulas. PACC-exe= preclinical Alzheimer’s cognitive composite plus additional measures of executive functioning.

Following a three-month washout period (at month 15), statistically significant differences emerged between groups for three of the seven cognitive measures, including the primary cognitive outcome. The MLI+EGCG group showed superior performance compared to the MLI+placebo group on the PACC-exe Z score (AMD: 0.19; 95% CI: 0.06, 0.32; p = 0.005; Hedges' g = 0.56), the Memory Composite Z score (AMD: 0.24; 95% CI: 0.04, 0.44; p = 0.022; Hedges' g = 0.51), and the Semantic Fluency Test (AMD: 2.5; 95% CI: 0.70, 4.3; p = 0.007; Hedges' g = 0.50).

### Safety Outcomes

The safety and tolerability of EGCG was good, with mainly mild adverse events over the 15 months of follow-up, unrelated to the treatment, and no differences between groups (Supplement 1, Appendix 2, Table 2, Table 3). Of 129 participants included, nine (7.0%) reported SAEs: three in the NRG (12.0%), five in the MLI+EGCG group (9.6%), and one in the MLI+placebo group (2.0%). Of 52 participants in each MLI group, 36 (69.2%) had 63 AEs in the MLI+EGCG group, and 40 (76.9%) had 58 AEs in the MLI+placebo group. The most common AEs were infections and infestations (25.0% vs. 21.6%), metabolism and nutrition disorders (23.1% vs. 15.7%), and blood and lymphatic system disorders (11.5% in MLI+EGCG vs. 15.7% in MLI+placebo). No withdrawals were related to drug tolerability.

### Secondary and Exploratory Outcomes

The MLI+EGCG intervention, compared to the MLI+placebo, did not affect brain structure and blood AD biomarkers (Supplement 1, Appendix 2, Table 4). Concerning dementia risk scores, anthropometric factors, and cardiovascular risk factors, no statistically significant differences between the MLI+EGCG and the MLI+placebo groups were observed after 12 months of intervention. However, insulin resistance evaluated with the HOMA-IR showed greater improvements in the MLI+EGCG (Hedge‘effect size of differences of 0.36). A mild increase in homocysteine levels (AMD: 0.83; 95% CI: -0.24, 1.42; p = 0.006). was observed in the MI+EGCG group, which decreased in the MI+placebo (Supplement 1, Appendix 2, Table 5). MedDiet adherence showed greater improvements in the MLI+EGCG than in the MLI+placebo, particularly after 3 months of treatment discontinuation (Supplement 1, Appendix 2, Table 6). Both MLI groups improved physical fitness as evaluated with the senior fitness test battery (Supplement 1, Appendix 2, Table 7). However, the MLI+EGCG group experienced greater improvements in the quality-of-life environmental health domain than the MLI+placebo group (Supplement 1, Appendix 2, Table 8).

### Exploratory analysis of cognitive and dementia risk outcomes with the NRG

After 12 months of intervention, both MLI groups showed greater cognitive improvement than the NRG, which received healthy lifestyle recommendations. The MLI+EGCG group demonstrated a statistically significant advantage over the NRG in PACC-exe Z scores (AMD: 0.28; 95% CI: 0.11, 0.45; p = 0.001), whereas the difference between the MLI+placebo group and the NRG did not reach significance (AMD: 0.17; 95% CI: -0.01, 0.34; p = 0.060). Compared to the NRG, the MLI+EGCG group also showed improvements in the Memory Composite (AMD: 0.29; 95% CI: 0.00, 0.58; p = 0.048) and Executive Function Composite (AMD: 0.20; 95% CI: 0.02, 0.38; p = 0.027), while the MLI+placebo group improved in executive function as well (AMD: 0.18; 95% CI: 0.001, 0.36; p = 0.048).

At month 15, following a three-month washout period, the MLI+EGCG maintained a significant advantage in PACC-exe scores compared to the NRG (AMD: 0.20; 95% CI: 0.01, 0.38; p = 0.033).

In terms of dementia risk, both MLI groups showed significant reductions in LIBRA index scores relative to the NRG (MLI+EGCG vs. NRG: -1.20 points; 95% CI: -2.16, -0.24; p = 0.012; MLI+placebo vs. NRG: -0.99 points; 95% CI: -1.98, -0.01; p = 0.049).

### Adherence

Adherence to intervention components was high: average participation of 89.9% for nutrition, 62.4% for gymnasium classes, 72.0% for cognitive training, and 79.1% for psychoeducation sessions, and remained high during all the follow-ups (Supplementary material, Appendix 2, Table 9 and Figure 1). Compliance with EGCG was 90.0% at six months and 85.1% at 12 months (Supplementary material, Table 10).

## Discussion

In agreement with the FINGER 2.0 Model, the PENSA Study suggests that combining a MLI with the dietary compound EGCG may be associated with cognitive improvements in *APOE*-ɛ4 carriers with SCD, with indications of long-term benefits. While cognitive improvements in MLI+EGCG compared to MLI+placebo were not statistically significant at 12 months, the trend observed (p=0.061) and the higher odds of achieving a reliable cognitive improvement suggest that this combination could potentially offer greater cognitive benefits compared to MLI alone, with implications for sustained cognitive health. Notably, after a 3-month treatment discontinuation, MLI+EGCG maintained significant cognitive advantages over MLI+placebo, indicating potential sustained effects of the intervention. Moreover, while statistical significance was not consistently achieved in comparisons between MLI+EGCG and MLI+placebo, results tended to numerically favor the MLI+EGCG group across most cognitive outcomes, supporting the hypothesis of an additive benefit. However, further research is necessary to confirm these findings in larger and longer trials and explore the potential mechanisms underlying EGCG's effects.

Moreover, although the observed cognitive gains were modest, they were achieved through a comprehensive, multidomain lifestyle intervention sustained over a 12-month period. This suggests a potentially favorable benefit-to-effort ratio, especially if such effects can be maintained or amplified in the longer term. Importantly, the treatment with EGCG was safe, demonstrated excellent adherence, and its cognitive impact gained significance after treatment discontinuation.

The focus on *APOE*-ɛ4 carriers with SCD provides valuable insights into early AD prevention in high-risk populations, addressing a critical need in precision prevention. This study is among the first to exclusively target this well-defined group, addressing a critical gap in the AD precision prevention landscape. Moreover, the individualized approach used in the MLI, tailored to the participants' needs, aligns with the growing emphasis on personalized medicine and highlights the potential for scalable, real-world applications of personalized AD prevention strategies.

The effect size estimates observed between the MLI+EGCG and MLI+placebo groups in cognitive performance are higher than those reported in similar multimodal prevention trials (7,9,37,38), highlighting the potential added value of EGCG supplementation. Specifically, the effect size for the primary cognitive outcome (PACC-exe) at 12 months reached 0.36, increasing to 0.56 after a 3-month treatment discontinuation. However, determining clinical relevance in cognitively unimpaired individuals remains challenging, as the PACC was designed to sensitively detect changes in cognitive function regardless of their clinical significance (33). To address this challenge, we employed reliable change indexes (RCI) as an exploratory approach to examine individual-level change patterns and provide a statistical foundation for meaningful change— a necessary but not sufficient condition for clinical significance. The MLI+EGCG group showed a greater proportion of participants with reliable cognitive improvements compared to the MLI+placebo group. These results, coupled with positive benefits on quality of life (environmental health domain), provide preliminary evidence that the observed group-level effects may translate into meaningful benefits at the individual level.

To further contextualize these findings, exploratory comparisons with a NRG receiving general healthy lifestyle advice showed greater cognitive improvements and reduced dementia risk in both MLI groups, supporting the robustness of the observed effects**.** Although these results should be interpreted cautiously due to the lack of randomization, and the observational nature of the comparison, and may be influenced by non-specific factors (e.g. increased attention and time dedicated to the active groups), they help to clarify the potential impact of the interventions beyond normal age-related cognitive trajectories. Aside from cognitive benefits, both MLI groups also showed within-group improvements in several aspects such as dementia risk scores (e.g. LIBRA and CAIDE index), cardiovascular risk factors (e.g. systolic blood pressure, LDL-cholesterol, triglycerides, glucose and fibrinogen) and lifestyle factors (e.g. MedDiet adherence and functional fitness). The pre-post improvements observed in both MLI groups reinforce the value of multimodal lifestyle interventions for addressing modifiable risk factors, supporting a broader, multifactorial approach to dementia prevention.

Several mechanisms could potentially explain the cognitive effects of EGCG. In the present study, changes in homocysteine a surrogate biomarker of EGCG activity, observed in the MLI+EGCG arm due to its interaction with the one carbon metabolism cycle, already demonstrate that the amounts of EGCG in the body sufficed to induce homocysteine alterations (36). Thus, the significant increase in plasma homocysteine concentrations in the MLI+EGCG group, although non-clinically relevant, indicate EGCG activity in the methyl cycle via inhibition of the kinase activity of DYRK1A (39), which phosphorylates tau and amyloid precursor protein (APP). In fact, pharmacological inhibition of DYRK1A reduces Aβ and tau pathology and improves AD-related cognitive impairments in mouse models of AD, delaying the onset of both Aβ plaques and tau-containing neurofibrillary tangles (NFTs) (40). However, given the absence of differences in plasma AD biomarkers between groups, it could be hypothesized that the effects of the MLI+EGCG on cognition are not exerted via amyloid and/or tau pathways in the one-year intervention period but rather though other modifiable risk factors for dementia, not specific to AD. The observed trend in the reduction in insulin resistance in the MLI+EGCG group, compared to the MLI+placebo, could support the activity of EGCG on glucose homeostasis (41), which is crucial given the impact of alterations in glucose homeostasis on brain health and dementia risk (42). Moreover, EGCG can permeate the blood-brain barrier, potentially promoting neurite outgrowth and restoring proteins involved in synaptic and neural plasticity-related pathways (19). Importantly, interventions that may reduce the risk of cognitive decline via AD-independent pathways will become increasingly important in the future, particularly when considering the availability of anti-amyloid drugs that could be complemented with preventive strategies acting via other pathways. This highlights the contribution of this study to the emerging field of precision prevention, by combining lifestyle interventions with bioactive nutraceuticals to address a wide range of risk factors.

The findings of this study may have significant public health implications. Although further research is needed to confirm and expand upon these results, the observed cognitive improvements suggest that an MLI combined with EGCG could contribute to preventive strategies in high-risk populations. If replicated in larger and more diverse samples, such interventions could potentially reduce the incidence of dementia and alleviate the burden on healthcare systems. Moreover, adapting these interventions to include eHealth technologies and community-based resources can enhance their accessibility and effectiveness across diverse geographic and demographic settings, promoting wider adoption in public health policies aimed at Alzheimer's prevention.

This study is not without limitations. The PENSA study was conceived as a proof-of-concept trial due to funding and deadlines for reporting results constraints. This limits the number of subjects included and the length of the intervention (one year). Additionally, the results from the NRG receiving healthy lifestyle recommendations that were not randomized are exploratory and not powered for direct comparisons. Priority was placed on randomizing participants within the MLI groups to examine the impact of adding EGCG to a well-established MLI, previously shown to benefit cognition in the FINGER study. The inclusion of this NRG could introduce potential biases, as these participants could differ in motivation and other characteristics compared to those in the randomized MLI groups. Second, the in-person delivery of the MLI in an urban setting and recruitment through a web-based system may limit scalability and have preferentially attracted more educated, tech-savvy participants, potentially affecting the generalizability of the results. Future studies should consider alternative recruitment methods and explore adaptations of the MLI that leverage eHealth technologies and community-based resources to facilitate implementation in diverse demographic and geographic settings, enhancing the applicability of the results to broader populations at risk for Alzheimer’s disease. Third, the selection of FontUp capsules for EGCG supplementation was based on their demonstrated efficacy and safety in previous studies, with local production ensuring logistics and quality control. While other brands might yield similar results, FontUp's formulation produced following GMPs ensures reproducibility. In summary, further research is needed to generalize the PENSA study findings in larger populations with different risk profiles, such as individuals with lower education level, or carrying other *APOE* genotypes. Future research should also examine the longer-term effects of the MLI+EGCG.

## Conclusion

In conclusion, the PENSA study, a proof-of-concept RCT conducted within the WW-FINGERs network (6), represents a novel approach to exploring the potential cognitive benefits of combining multimodal lifestyle interventions with bioactive supplements like EGCG in individuals at high risk of developing AD. Cognitive improvements in MLI+EGCG compared to MLI+placebo at 12 months approached statistical significance (p=0.061), and by 15 months, the MLI+EGCG group demonstrated significant cognitive benefits, suggesting promising long-term effects of the intervention. Further research is needed to validate the observed effects and elucidate the mechanisms that drive cognitive changes, determine the optimal intensity and dosage of intervention required to impact cognitive performance, identify factors that affect adherence and cognitive response, and evaluate the intervention's effectiveness in the longer term and across various settings and populations.

## Funding sources

This study was mainly funded by the Alzheimer’s Association, The PART THE CLOUD to RESCUE (REverse, reStore, Cease and UndErstand) Brain Cell Degeneration in Alzheimer’s disease Program, grant 18PTC-R-592192 and Project PI17/00223, funded by Instituto de Salud Carlos III (ISCIII) and co-funded by European Regional Development Fund (FEDER). RdlT leads the Consolidated “Clinical Research Group in Pharmacology and Development of Biomarkers and New Drugs”, supported by the Generalitat de Catalunya (2021 SGR 00253 and 2021 SGR 01421). This research was also supported by CIBER-Consorcio Centro de Investigación Biomédica en Red- (CB06/03/0028), Instituto de Salud Carlos III (ISCIII), Ministerio de Ciencia, Innovación y Universidades (PID2022-141900OB-I00; PID2023-148033OB-C21) and Unión Europea – FEDER Fund; Grant AC22/00004 funded by Instituto de Salud Carlos III, and by the European Union NextGenerationEU, Mecanismo para la Recuperación y la Resiliencia (MultiMemo, JPND program); and by the "Responsible Research and Innovation Awards" (RRI20/00005) organized by the ISCIII and promoted within the framework of the European project ORION (Open Responsible research and Innovation to further Outstanding kNowledge). NS-D had a predoctoral fellowship (2022 FI_B1 00026) from AGAUR-Generalitat de Catalunya, and TL had a predoctoral fellowship FI18/00041 from the ISCIII. The funding organizations had no role in the design and conduct of the study; collection, management, analysis, and interpretation of the data; preparation, review, or approval of the manuscript; or decision to submit the manuscript for publication.

## CRediT authorship contribution statement

**Laura Forcano:** Writing – review & editing, Writing – original draft, Project administration, Investigation, Conceptualization. **Natalia Soldevila-Domenech:** Writing – review & editing, Writing – original draft, Methodology, Investigation, Formal analysis. **Anna Boronat:** Writing – review & editing, Investigation. **Gonzalo Sánchez-Benavides:** Writing – review & editing, Investigation. **Albert Puig-Pijoan:** Writing – review & editing, Investigation. **Thais Lorenzo:** Writing – review & editing, Investigation. **Ana Aldea-Perona:** Writing – review & editing, Supervision, Investigation. **Marc Suárez-Calvet:** Writing – review & editing, Investigation. **Aida Cuenca-Royo:** Writing – review & editing, Investigation. **Juan Domingo Gispert:** Writing – review & editing, Investigation. **Maria Gomis-Gonzalez:** Writing – review & editing, Investigation. **Carolina Minguillón:** Writing – review & editing. **Patrícia Diaz-Pellicer:** Writing – review & editing, Investigation. **Karine Fauria:** Writing – review & editing. **Iris Piera:** Investigation. **Klaus Langohr:** Writing – review & editing, Writing – original draft, Formal analysis, Data curation. **Mara Dierssen:** Writing – review & editing, Investigation. **Nieves Pizarro:** Writing – review & editing, Investigation, Funding acquisition, Conceptualization. **Esther Mur-Gimeno:** Writing – review & editing, Investigation. **Oriol Grau-Rivera:** Writing – review & editing, Writing – original draft, Resources, Investigation. **José Luis Molinuevo:** Writing – review & editing, Funding acquisition, Conceptualization. **Rafael de la Torre:** Writing – review & editing, Writing – original draft, Supervision, Investigation, Funding acquisition, Conceptualization.

## Declaration of Competing interests

The authors declare the following financial interests/personal relationships which may be considered as potential competing interests:

Jose Luis Molinuevo reports a relationship with H Lundbeck that includes: employment. If there are other authors, they declare that they have no known competing financial interests or personal relationships that could have appeared to influence the work reported in this paper.

## Appendix Supplementary materials

Image, application 1
