# Supplementary material for: A multimodal lifestyle intervention complemented with epigallocatechin gallate to prevent cognitive decline in APOE- ɛ4 carriers with Subjective Cognitive Decline: a randomized, double-blinded clinical trial (PENSA study)
Source: J Prev Alzheimers Dis. 2025 Jul 15;12(8):100271. doi: 10.1016/j.tjpad.2025.100271 (PMC12413734; doi:10.1016/j.tjpad.2025.100271)
Supplement: Supplementary file 1 [file mmc1.docx]

**Supplement 1**

Forcano L, Soldevila-Domenech N, Boronat A, et al. A multimodal lifestyle intervention complemented with epigallocatechin gallate to prevent cognitive decline in *APOE*- ɛ4 carriers with Subjective Cognitive Decline: a randomized, double-blinded clinical trial (PENSA study)

[1. Appendix 1. Detailed study methods 2](#_Toc198038946)

[1. Randomization procedure and masking 2](#_Toc198038947)

[2. Exclusion criteria and rules for termination of study 2](#_Toc198038948)

[3. Design of the study and schedule of assessments 2](#_Toc198038949)

[4. Sample Size 4](#_Toc198038950)

[5. EGCG treatment 4](#_Toc198038951)

[6. Description of the multimodal intervention components 4](#_Toc198038952)

[6.1. Diet 4](#_Toc198038953)

[6.2. Physical activity 5](#_Toc198038954)

[6.3. Cognitive training 6](#_Toc198038955)

[6.4. Psychoeducation group sessions 9](#_Toc198038956)

[6.5. Social stimulation group sessions 9](#_Toc198038957)

[7. Neuropsychological study battery 9](#_Toc198038958)

[8. Primary, secondary and exploratory efficacy measures 11](#_Toc198038959)

[9. MCI diagnosis 11](#_Toc198038960)

[10. Safety parameters definitions 12](#_Toc198038961)

[11. Baseline characteristics 12](#_Toc198038962)

[12. Magnetic Resonance Imaging (MRI) 12](#_Toc198038963)

[13. Blood AD biomarkers 13](#_Toc198038964)

[14. Dementia risk scores and cardiovascular risk factors 13](#_Toc198038965)

[15. Olfactory functioning, lifestyle and physical functioning measures 13](#_Toc198038966)

[16. Functionality measures 14](#_Toc198038967)

[17. Adherence to the multimodal intervention 15](#_Toc198038968)

[18. Online-Only References 15](#_Toc198038969)

[2. Appendix 2. Supplementary Results 18](#_Toc198038970)

[2.1. Supplementary Tables 18](#_Toc198038971)

[Table 1. SCD characteristics of all included participants. 18](#_Toc198038972)

[Table 2. Self-reported adverse effects (as ‘System Organ Class Terms’) by treatment group during the 15 months of follow-up in included participants. 19](#_Toc198038973)

[Table 3. Self-reported adverse effects (as ‘Preferred Terms’) by treatment group during the 15 months of follow-up in included participants. 20](#_Toc198038974)

[Table 4. Intervention effects on brain structure (MRI) and plasma AD biomarkers 22](#_Toc198038975)

[Table 5. Intervention effects on dementia risk scores, anthropometric factors and cardiovascular risk factors. 23](#_Toc198038976)

[Table 6. Intervention effects on lifestyle risk factors. 25](#_Toc198038977)

[Table 7. Intervention effects on functional fitness evaluated with the Senior Fitness Test battery. 26](#_Toc198038978)

[Table 8. Intervention effects on quality of life, mental health, and adaptive behavior. 27](#_Toc198038979)

[Table 9. Adherence to MLI components. 28](#_Toc198038980)

[Table 10. Determination of EGCG in plasma as an indicator of treatment compliance. 29](#_Toc198038981)

[2.2. Supplementary figures 30](#_Toc198038982)

[Figure 1. Monthly adherence to the multimodal intervention components. 30](#_Toc198038983)

[3. The PENSA Study Working Group 31](#_Toc198038984)

*This supplemental material has been provided by the authors to give readers additional information about their work.*

# Appendix 1. Detailed study methods

## Randomization procedure and masking

An independent statistician (KL) generated randomization sequences separately for female and male participants in blocks of four (two of each sex) using the function ‘sample’ of the statistical package R. Randomization sequences were uploaded into the electronic Case Report Form (eCRF), which automatically assigned a treatment group to each participant upon inclusion. Exclusive access to treatment allocation was granted to the Pharmacy Department of Hospital del Mar, responsible for provisioning identical presentations of active and placebo treatments.

Outcome evaluators, blinded to the MLI arms or NRG, were not involved in intervention activities. Participants were instructed not to discuss the intervention with outcome evaluators. The double blind was maintained until the study eCRF was closed. Statistical analyses were done without unblinding the multimodal intervention (MLI) groups.

## Exclusion criteria and rules for termination of study

Exclusion criteria included: (i) history of neurological or psychiatric conditions according to DSM-5 criteria; (ii) clinically significant abnormalities in laboratory test; (iii) any contraindication for brain magnetic resonance imaging (MRI); (iv) presence of mild to moderate leukoaraiosis (scoring 3 on Fazekas scale, and/or less than three lacunar infarcts not localized on strategic brain regions (e.g. bilateral thalamic); (v) primary or recurrent malignant disease treated within the last 2 years; (vi) evidence of medical conditions/medications that may interfere with study assessments; and (vii) current intake of vitamins, products containing EGCG supplements for at least 3 months previous to the screening visit.

Rules for early termination of the study were predefined and included: (i) observation of severe adverse events that did not permit the trial to be continued; (ii) undesirable events that, according to the opinion of the principal investigator, were considered as hazardous for the physical and psychical health of the individual participant when continuing the trial; (iii) clinically significant measured data that constitute participant exclusion criteria and/or indicate harm to the investigational participant’s health by medical knowledge; (iv) violation of the trial protocol; (v) non-compliance; (vi) withdrawal of consent by the investigational participant; and (vii) difficulties arising from administrational problems.

## Design of the study and schedule of assessments

**
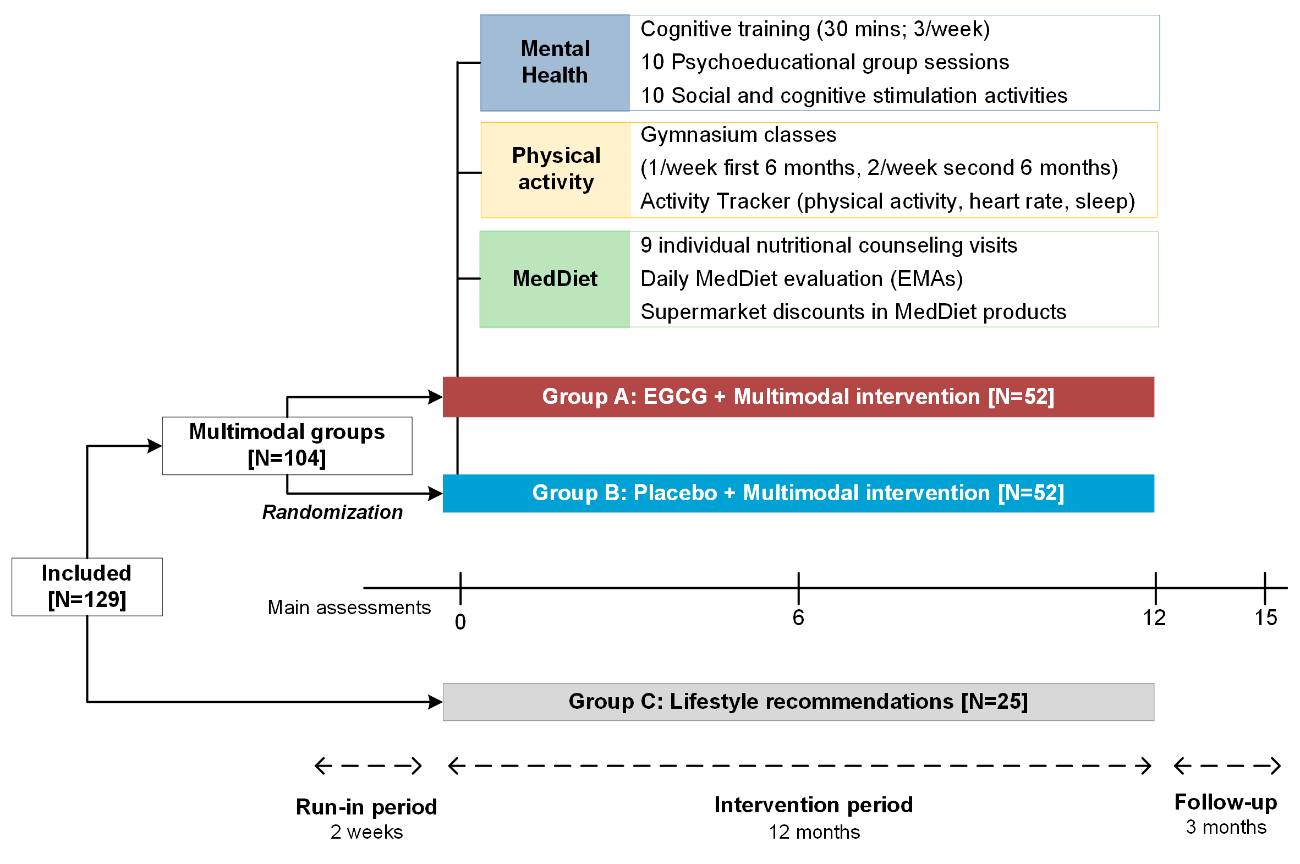
**

**Figure S1. Schematic diagram of the PENSA Study design**

**Table S1. Schedule of assessments**

| **Period** | **Pre-screening** | **Screening** | **RZ** | **Run-in** | **Treatment** | | | **Follow-up** |
| --- | --- | --- | --- | --- | --- | --- | --- | --- |
|  |  |  |  |  |  |  |  |  |
| **Procedure/ Month** |  |  |  | **M**  **-1** | **M**  **0** | **M6** | **M**  **12** | **M**  **15** |
| Ad-hoc internet-based SCD questionnaire | x |  |  |  |  |  |  |  |
| *APOE* genotyping and communication | x |  |  |  |  |  |  |  |
| SCD-Plus criteria |  | x |  |  |  |  |  |  |
| Medical interview and neurological exploration |  | x |  |  |  |  | x |  |
| Clinical exam^a^, vital signs^b^ and adverse events |  | x |  |  | x | x | x | x |
| ECG, blood chemistry, hematology and coagulation^c^ |  | x |  |  |  | x | x |  |
| PACC-exe and additional neuropsychological tests |  | x |  |  |  | x | x | x |
| Neuroimaging acquisitions (MRI, fMRI) |  | x |  |  |  |  | x |  |
| Safety assessment of EGCG and biomarkers of treatment compliance |  |  |  |  |  | x | x |  |
| Blood sampling for biomarkers^d^ and safety parameters^e^ |  |  |  |  | x | x | x |  |
| Genetics and epigenetics |  |  |  |  | x |  | x |  |
| Urine sampling for dietary patterns (metabolomics) |  |  |  |  | x | x | x |  |
| Fecal sampling for microbiota |  |  |  |  | x | x | x |  |
| Plasma neuron-derived exosomes and oral fluid |  |  |  |  | x |  | x |  |
| Olfactory function |  |  |  |  | x |  | x |  |
| Adaptive behavior |  |  |  |  | x | x | x | x |
| Quality of life, mental and general health |  |  |  |  | x | x | x | x |
| Lifestyle habits (questionnaires) |  |  |  |  | x | x | x | x |
| Informative session  (separately for MLI and NRG) |  |  |  | x |  |  |  |  |
| Revision of adverse events |  |  |  |  |  | x | x | x |
| Functional improvement |  |  |  |  | x |  | x |  |
| Lifestyle habits (continuous assessments)^f^ |  |  |  |  | | | | |
| SCD= Subjective cognitive decline; RZ = randomization; AD= Alzheimer’s disease; MLI= Multimodal Intervention; NRG= Non-randomized Group.  a. Physical examination, height, weight and BMI.  b. BP, HR and temperature. BP and HR should be measured prior blood draws if possible, in supine position.  c. Biochemical: Glycosylated Hemoglobin (HbA1c), vitamin B12, Folate, TSH, T4, Insulin, creatinine, urea, glucose, urate, sodium, potassium, chlorine, albumin, ferritin, lactate dehydrogenase (LDH), creatine phosphokinase (CPK), alkaline phosphatase (ALP), aspartate aminotransferase (SGOT/AST), alanine aminotransferase (SGPT/ALT), gamma-glutamyl transferase (g-GT), total bilirubin, calcium, total proteins, total cholesterol, cholesterol HDL, cholesterol LDL, triglycerides. Hematology: Hemoglobin, hematocrit, reticulocytes (absolute), RBC, platelet count, WBC and differentials (absolute), MCV, HCM, CHCM, RDW-CV, VPM. Coagulation: Activated partial thromboplastin time (aPTT or APTT), aPTT ratio and prothrombin time (PT), INR, fibrinogen. Serology: Serum Hepatitis B virus surface antigen (HBSAg), Antibodies IgG virus Hepatitis C serum, Human serum immunodeficiency virus antibodies (VIH), AC Treponema pallidum SE. Urine: Urianalysis (density, pH, glucose, ketone bodies, bilirubin, urobilinogen, proteins, red blood cells, leukocytes, nitrites); urinary sediment.  d. Superoxide dismutase (SOD), Glutathione reductase (GR), NADPH- Quinone Oxidoreductase 1 (NQO1) Heme oxygenase-1 (HO-1) + ageing biomarkers (epigenetics), (Aβ1-42),(T-tau),(P-tau181P), light-NFL, ICAM-1, VCAM-1  e. Creatinine, urea, ionogram, spartate aminotransferase (SGOT/AST), alanine aminotransferase (SGPT/ALT), gamma-glutamyl transferase (g-GT), total bilirubin, TSH and glycosylated hemoglobin (HbA1c).  f. Activity tracker measures and ecological momentary assessments | | | | | | | | |

## Sample Size

We calculated sample size by estimating the minimum difference in the change of the PACC-exe score between groups, considering previous observational studies involving clinically normal elderly individuals with evidence of AD pathology and *APOE*-ε4 carriers.^23^ Anticipating a difference of roughly 1.5 units (SD 2.4) between groups, and assuming a correlation of 0.5 between visits, we estimated that 45 participants in each group would be needed for a significance level of 5% and a power of 80%, with a dropout rate of 10%. To ensure robustness, we aimed to include 50 participants in each MI group.

## EGCG treatment

EGCG treatment was administered orally in a capsule format. Capsules of 345 mg contained maltodextrin (i.e., placebo, supplied by Laboratori Farmàcia Eixample) or 225.5 mg of decaffeinated green tea extract (i.e., active treatment) of which 100 mg were EGCG (min 94% purity of EGCG), supplied by Laboratoires Grand Fontaine (FontActiv^®^, FontUp capsules). A dose of approximately 5-6 mg/kg up to a maximum 500 mg/day was administered to participants for 12 months. EGCG dosage was established by body weight ranges (300 mg/day for participants up to 50 kg and 500 mg/day for participants over 50 kg). Only four participants (two with active treatment and two with placebo) received the low dosage of 300 mg/day. Participants took three or five capsules/day depending on the dosage: one/two before breakfast, one/two before lunch, and one before dinner, respectively. Same amounts of placebo preparation were taken by participants in the multimodal + placebo group.

## Description of the multimodal intervention components

## Diet

Individually tailored dietary recommendations to follow a traditional MedDiet were given at the beginning of the study based on the dietary habits observed during the run-in period (during which two 3-day food diaries were administered). Recommendations were also based on participants’ nutritional status, comorbidities (obesity, hypertension, diabetes, and hyperlipidemia), and cooking skills (**Table S2**). The energy intake of the diet was adjusted to the energy expenditure to achieve a healthy weight. In general, the nutritional profile of the MedDiet prescribed was as follows: 30-35% of the energy as fat (<7% of saturated fatty acids, >20% of monounsaturated fatty acids and >10 % polyunsaturated fatty acids), 15% of the energy in the form of protein and 50-55% as carbohydrates. There were nine individual nutritional counselling sessions (at baseline and after 0.5, 1, 1.5, 2, 3, 4, 6 and 9 months) that aimed to reinforce, revise and readjust the intervention and its progression. In those visits, participants were either asked to bring a 3-day food diary or a 24h dietary recall was performed. With this information, the nutritionist adjusted the intervention. MedDiet adherence was weekly monitored throughout the study using daily ecological momentary assessments (EMAs)^1^, and individualized reports were monthly delivered to participants with dietary strategies to improve MedDiet adherence. Participants also benefitted from supermarket discounts in selected MedDiet products.

**Table S2. Dietary recommendations.**

| **Basic MedDiet recommendations** | ***Every main meal:*** | Fruit: 1 - 2 servings  Vegetables ≥ 1 servings  Extra virgin Olive oil |
| --- | --- | --- |
|  | ***Every day:*** | Dairy: 2 servings (preferably low in fat)  Nuts or seeds: 1 - 2 servings  Herbs, species, ... (instead of salt) |
|  | ***Every week:*** | White meat: 2 servings  Fish and Seafood: ≥ 2 servings  Eggs: 2 - 4 servings  Pulses: ≥ 3 servings  Red meat: ≤ 1 servings  Processed meat ≤ 1 serving |
|  | ***Drink:*** | Water is the drink of choice  Moderate red wine consumption is allowed |
|  | ***Selection of products and baking:*** | Selection of seasonal products  Variety of colors and textures  Simple cooking: boiled, grilled, steamed... avoid stews and deep frying  Complex carbohydrates: 1 - 2 servings (Bread, pasta, rice, couscous or other cereals, preferably whole grains) |
| **Personalized MedDiet recommendations to comorbidities** | ***Unstable weight for the last three months (>3 kg)*** | Assess the nutritional risk |
|  | ***BMI >25 kg/m^2^*** | Hypocaloric MedDiet |
|  | ***BMI < 18.5 kg/m^2^*** | Assess the nutritional risk, and promote the intake of specific products to prevent malnutrition |
|  | ***Type 2 diabetes or glucose intolerance*** | Weight reduction if necessary  Sodium intake <2400 mg/day  Promote fiber intake  Limit the intake of saturated and trans fatty acids  Promote foods with a low glycemic index and limit simple sugars  Increase consumption of n3-PUFA |
|  | ***Hypercholesterolemia*** | Limit the intake of saturated and trans fatty acids  Increase consumption of n3-PUFA  Promote the consumption of fiber |
|  | ***Hypertriglyceridemia*** | Suppress the intake of simple sugars  Limit alcohol intake  Increase consumption of n3-PUFA  Maximum fruit consumption of 700g/day |
|  | ***Hypertension*** | <2400 mg/day of sodium  Promote fiber intake  Promote consumption of foods rich in calcium, potassium and magnesium |
| **Other considerations** | ***Limited budget*** | Proposals/ideas for low-budget MedDiet dishes |
|  | ***Limited cooking skills*** | Recommendations of easy-preparing healthy plates |

## Physical activity

Participants were invited to participate in group-based 60-minute physical activity sessions combining aerobic, strength, and balance activities (minimum of one session/week the first 6 months, and two sessions/week from month 7 to 12) in a gymnasium. Sessions were personalized according to participants’ baseline physical condition status and were guided by expert sport coaches (**Table S3**). Participants also received a wristband activity tracker (Models: Fitbit^®^ Inspire 2 or Charge 2: Fitbit Inc, San Francisco, California) for use during the run-in and treatment periods, which can estimate steps, distance, calories, active minutes, heart rate, and sleep duration and stages. Besides monitoring the physical activity of participants, Fitbit data was also used to deliver monthly personalized recommendations for increasing the amount of aerobic exercise. The first short-term goal was to achieve a physically active lifestyle according to the classification system based on steps-per-day categories^2^. Healthy older adults should take 10,000 steps/day, and individuals living with disability or chronic illness should achieve about 8,500 steps/day. The use of steps was expected to increase the motivation of participants to produce a behavioral change, as it is an intuitive and easily understandable measure^3^. The second goal was to reach the minimum recommendations of moderate physical exercise in older adults, which are 150-210 minutes/week (90-150 if history of cardiovascular disease, osteoporosis or mobility problems such as osteoarthritis)^4^. This goal was evaluated with the number of active minutes per week registered with the Fitbit. This device considers active minutes when physical activity is performed at an intensity of 3 or more metabolic equivalent tasks (METs) at least 10 minutes in a row^5^.

**Table S3. General structure of the gymnasium sessions.**

| **Structure** | **Duration** | **Components** | **Exercises** |
| --- | --- | --- | --- |
| Warm-up | 10-15 min | Game-based exercises to promote social and physical interactions, prevent joint and muscle injuries and increase heart rate progressively. | Global mobility exercises, dynamic stretches of the major muscle groups, and exercises of coordination and balance. Exercises of low intensity and with few repetitions. |
| Main part | 35-40 min | Aerobic fitness, muscle endurance and strength, coordination and rhythm. | Aerobic phase with exercises based on games that involve moving around the room at different speeds. Training circuits with different stations aimed at training muscle strength and endurance with self-loads, which progressively increased in the number of repetitions and in the execution speed. Circuits were based on functional exercises such as stair climbing, rising from a chair, carrying heavy bags or sprints “to catch a bus”. Such circuits were organized controlling the time in each station, with consecutive stations and interspersing cardiovascular training between stations. Elastic bands and free weights were often used. Some sessions also included choreographies.  The training intensity of aerobic and strength exercises was of 4-6 during the first 2 months and 7-9 during the following months, according to the modified Borg Scale of Perceived Exertion^6^ and adapted to each individual. |
| Cool-down | 10-15 min | Balance-based functional exercises, flexibility exercises and breathing exercises. | Static balance-based activities designed to challenge the vestibular (e.g., move head), visual (e.g., eyes open/closed) and somatosensory (e.g., stand on foam) systems, coordination exercises and passive stretches of different muscle groups. |

## Cognitive training

The cognitive training program was delivered through a telematic platform, NeuronUP^©^, that offers neurorehabilitation materials for cognitive stimulation to professionals. The cognitive training plan was designed by experienced neuropsychologists and included 36 different activities that covered different cognitive domains relevant to the AD’ related cognitive impairment profile (**Table S4**). These 36 activities were monthly distributed in 12 sessions (2-3 sessions/week) of about 30 minutes each. Therefore, each cognitive training session included 3 activities of about 10 minutes each that exercised different domains, and each activity was performed once/month. Participants completed the sessions remotely using their own computer or tablet. Details on the cognitive training intervention have been published previously^7^.

**Table S4. NeuronUP cognitive training program.**

| **Domain** | **Name** | | **Type/session** | | **Instructions** | |  |
| --- | --- | --- | --- | --- | --- | --- | --- |
| Memory | Press conference | | Game/1 | | Remember the order in which the journalists raise their hands and stand up and reproduce the exact same order | |  |
|  | Color lottery | | Game/6 | | Remember the color of balls that go into the lottery machine to later identify which colored ball comes out from the lottery machine | |  |
|  | Illuminated windows | | Game/3 | | Remember the position of the windows in the house to replicate it later (fixed memorization time) | |  |
|  | Matching the cards | | Game/8 | | Find the pairs among a set of cards that are face down | |  |
|  | Straighten up the kitchen | | Game/10 | | Place some ordinary kitchen items in their usual place | |  |
|  | Order the sequence | | Worksheet/11 | | Order a series of previously memorized visual elements | |  |
| Executive functions | Sorting bugs | | Game/4 | | Reorganize the moving elements (bugs) by placing each type on the side where they belong | |  |
|  | Balance the bags | | Game/7 | | Based on the weight of different products, put them into bags distributing their weight equally | |  |
|  | Home delivery | | Game/5 | | Remember the order in which the buildings light up to later reproduce it in reverse order | |  |
|  | Stop the ball | | Game/2 | | Calculate the exact moment when the element should pass through a specific place | |  |
|  | Card Pyramid | | Game/9 | | Arrange the cards that appear in either increasing or decreasing numerical order | |  |
|  | Knitting a scarf | | Game/8 | | Catch all the balls of yards that appear without hitting anything | |  |
|  | Jigsaw puzzle | | Game/5 | | Connect the pieces until they make a picture | |  |
|  | Déjà vu | | Game/11 | | Look closely at all the elements and find those appearing more than once | |  |
|  | Exact payments | | Generator/11 | | Select the exact amount of money requested | |  |
| Attention | Fast numbers | | Game/5 | | Find as quickly as possible the numbers that stay still among a group of numbers that are constantly changing | |  |
|  | Colored shapes | | Game/7 | | Look at a stimulus that is changing colors and react only when the indicated color appears | |  |
|  | The clone | | Game/10 | | Finding as quickly as possible the repeated element | |  |
|  | Collect your luggage | | Game/12 | | Select only the suitcases matching the model from a moving luggage set | |  |
|  | Word scramble | | Generator/1 | | Put the letters in the correct order to form words that make sense | |  |
|  | Sorting numbers | | Generator/2 | | Sort a sequence of moving numbers | |  |
| Language | | Jumbled letters I | | Game/4 | | Order letters to create a word | |
|  |  | Jumbled letters II | | Game/12 | | Order letters to create a word | |
|  |  | Making words | | Game/3 | | Make words by putting fragments, syllables or individual letters in the correct order | |
|  |  | Making sentences | | Game/10 | | Sort words to make coherent syntactic structures | |
|  |  | Naming objects | | Worksheet/2 | | Name objects with the help of phonological clues | |
|  |  | Matching noun with image | | Worksheet/6 | | Match each noun with the image that represents it. | |
| Visuospatial | | Organize the bookcase | | Game/6 | | Copy the position of the objects as shown in the model | |
|  |  | Intertwined | | Game/3 | | Make a geometric shape by moving its vertices so that none of its sides cross each other | |
|  |  | The neighbor’s garden | | Game/1 | | Determine the spatial correspondence of a stimulus | |
|  |  | Combining figures | | Game/9 | | Complete the figure as it would look as a result of joining different figures | |
|  |  | Angles | | Game/8 | | Complete the defined angle based on the reference line | |
|  |  | Cube movement | | Worksheet/11 | | Calculate what a series of cubes would look like after moving some of them | |
| Orientation | | Location in time | | Worksheet/7 | | Locate in time, day, month, year and season | |
|  |  | Objects, establishments and professionals | | Worksheet/9 | | Relate various objects with the places where they are obtained and the professionals who take care of them | |
|  |  | Home objects | | Worksheet/12 | | Locate where various objects would be in the house | |

## Psychoeducation group sessions

The psychoeducation program consisted of ten group sessions of 90 minutes (7 monthly and 3 bimonthly sessions), delivered by experienced psychologists, designed to motivate and empower participants to elongate the impact of the lifestyle intervention. Participants were taught about the nature of cognitive decline (genetic, lifestyle and environmental factors), behavioral change strategies, what to expect during and after the intervention, and how changes in lifestyle habits could help them in dealing with cognitive decline (**Table S5**).

**Table S5. Discussion topics of the psychoeducation group sessions**

| **Session** | **Discussion topic** |
| --- | --- |
| 1 | Presentation, promotion of mutual trust and motivational interview. Understanding normal ageing and cognitive decline |
| 2 | Developing a positive lifestyle: Physical activity |
| 3 | Developing a positive lifestyle: Healthy Diet |
| 4 | Developing a positive lifestyle: Brain Health |
| 5 | Learning strategies to elongate a healthy lifestyle |
| 6 | Social support: involving family and friends in your active lifestyle |
| 7 | Mid-treatment review: strengths and weaknesses |
| 8 | Problem-solving strategies |
| 9 | “Relapse” prevention strategies |
| 10 | Recapitulation of main concepts on active lifestyle and planning for future |

## Social stimulation group sessions

The social stimulation intervention consisted of ten 90-120 minutes sessions, led by specific professionals according to the specific topic of the session (e.g., museum visits, outdoor sport activities, cooking lessons, musical activities, mindfulness session, aqua gym session, cardiopulmonary resuscitation course, and other activities proposed by participants). The purpose of these sessions was to provide opportunities for participants to interact with their groupmates, as well as to provide them access to environments and resources that may help to keep themselves cognitively stimulated.

## Neuropsychological study battery

#### Memory

**Verbal episodic memory:** Measured with the Free and Cued Selective Reminding Test (FCSRT)^8^.The FCSRT consists of the learning and retention of a list of 16 semantically unrelated words through a controlled learning process that uses semantic encoding. First the participant is asked to read aloud the 16 words (4 words in 4 cards) and associate them with their corresponding semantic cue (learning phase). Then, three trials of 90s each are performed, each one preceded by 20s of a number subtraction task. Each trial consists of free recall followed by cued recall for the words not spontaneously retrieved, by using the semantic cues previously given. The words that are not recalled after cueing are selectively reminded in the two initial trials, but not in the last one. A delayed free and cued recall is performed after 25-35 min. The main variables of the test are: the sum of the words correctly retrieved in the three free recall learning trials [Immediate Free Recall (FCSRT-IFR; range 0–48)]; the sum of the words recalled, either free or cued, in the three immediate recall trials [Total Recall (FCSRT-TR; range 0–48)]; the delayed free recall [Delayed Free Recall (FCSRT-DFR; range 0–16)]; and the total amount of words recalled, either free or cued, in the delayed recall trial [Total Delayed Recall (FCSRT-TDR; range 0–16)]. The Spanish validated version A of the FCSRT was used in this study.

**Auditory memory:** Measured with the Logical Memory (LM), a sub-test from the Wechsler Memory Scale (WMS)^9^. The LM has three parts: LM I (immediate recall, LM-IR), LM II (delayed recall, LM-DR), and LM Recognition (delayed recognition, LM-recognition). In the LM I, subjects are required to immediately recall details of two short passages. In the LM II, subjects are asked to recall the passages after a 20 to 30-minute delay. In both parts, the memory score is computed by summing up the number of the remembered items for each story. Finally, in the LM Recognition, subjects are asked to answer yes/no questions regarding the passages learned earlier. In this study we used stories B and C regardless of age. LM-IR ranges 0-50, LM-DR history C ranges 0-25, LM-DR total ranges 0-50, and LM-recognition ranges 0-30 points.

#### Executive functioning

**Sustained attention and shifting**: Measured with the Coding test score from the Wechsler Adult Intelligence Scale–IV (WAIS-IV)^10^. It consists of a paper-and-pencil cognitive test presented on a single sheet of paper where the examinee is asked to fill in the correct symbols into the spaces below the numbers, by matching them according to a key located on the top of the page. The number of correct symbols within 120 seconds constitutes the total score.

**Inhibition and attention:** Measured with the Stroop Color and Word Test^11^. This test consists of three printed sheets with 100 words in each, distributed in 5 columns. Participants are allowed to read each sheet for 45 seconds and the total number of words read is recorded. Errors are discounted for the total of words in each part. Three scores are obtained: *W* (number of words correctly read in the first sheet), C (number of colors correctly named in the second sheet) and CW (number of items correctly named in the third sheet). An interference score was calculated by subtracting a predicted color-word value (*CW’*) from the obtained *CW* score, with higher scores reflecting lower difficulties in inhibiting interference^12^. *CW’* was calculated using the following formula: (((216 – *W*) × *C*) / ((216 – *W*) + *C*)).

**Working memory**: Measured with the Five Digit Test (FDT)^13^. It is a numerical Stroop task divided into four parts. In part 1 (Reading), subjects have to name numbers from 1 to 5 as fast as they can. In part 2 (Counting), they need to describe quantities from 1 to 5. Boxes contain asterisks and participants are asked to state the number of asterisks in each box. Part 3 (Focusing) involves a selective attention trial, where subjects must ignore the identity of numbers, but have to tell how many numbers are present in each stimulus, in an incongruent condition. In part 4 (Switching), an extra clue indicates whether the participant must state the number of digits or their identity (reading or counting). In each section of the test, performance is measured in terms of the time required to complete the task. FDT-Inhibition (Focusing minus Reading) and FDT-Flexibility (Switching minus Reading) scores are calculated to measure working memory components related to the executive system. Scores were reversed (multiplied by -1) so that higher Flexibility or Inhibition scores indicate better working memory performance.

**Semantic verbal fluency**: It entails the generation of words from a given category (animals) within a pre-set time of 60 seconds.^14^

**Attention and Working Memory**: Measured with the Digit Span subtest (DS) from the WAIS-IV^15^. It is a verbal task, with stimuli presented auditorily, and responses spoken by the participant and scored automatically by the software. Participants are presented with a random series of digits of increasing length (maximum of 9 digits), and are asked to repeat them in either the order presented (forward, DSF) or the inverse order (backwards, DSB). Then, participants are asked to sequence the digits without repetitions (sequencing span, DSS), as some of the longer sequences include repeated digits. DSF is a good measure of simple attention and short-term memory. DSB and DSS represent a qualitatively different type of task that relies more upon working memory skills.

#### Global cognition

Measured with The Mini Mental State Examination (MMSE)^16^ and the Montreal Cognitive assessment (MoCA)^17^. The MoCA is a screening test for detecting cognitive impairment with a high load of executive functioning tasks.

#### Expressive language

Measured with the reduced 15-item version of the Boston Naming Test (BNT)^18^. The subject is asked to name each object correctly within a maximum of 20s. Semantic or phonemic cues are provided when necessary. The final score is calculated by summing the number of items that are correctly named spontaneously plus additional items that are correctly named after semantic cues.

#### Perceptual reasoning

Measured with the Visual Puzzle Test from the WAIS-IV^15^. It consists of 26 puzzles that are presented complete to the examinee. The examinee is asked to select three pieces to build the presented puzzle.

## Primary, secondary and exploratory efficacy measures

The primary outcome measure is change in the PACC-exe score, a composite score based on the results from six tests, calculated as Z scores standardized to the baseline mean and SD, with higher scores suggesting better performance. Specifically, it includes measures of short-term verbal episodic memory (the FCSRT-TR), long-term auditory memory (the LM-DR history C), sustained attention and shifting (the Coding test total score), global cognition (the MoCA total score), inhibition and attention (the Stroop interference score) and working memory (the FDT-flexibility score; reversed). The MoCA was chosen over the MMSE due to its higher sensitivity to subtle and early cognitive changes in older adults without dementia.

Details on safety and tolerability assessments: AEs and SAEs reported by participants or identified through protocol-specified safety laboratory assessments including thyroid, liver, and renal function, and vital signs measurements. These events were meticulously documented by the study physicians, categorized, and reported using preferred terms and s. AEs and SAEs were categorized and reported using preferred terms and system organ class terms according to their etiology, manifestation site, or purpose, ensuring a comprehensive evaluation of safety.

Secondary endpoints included changes in structural magnetic resonance imaging (MRI), assessing hippocampal and lateral ventricular volumes, cortical thickness, and white matter hyperintensities (WMH). T1-weighted scans were segmented with Freesurfer version 7.1 and parcellated according to the Desikan-Killiany atlas. This included scans of hippocampal and lateral ventricles, with cortical thickness composites computed for brain regions known to suffer from atrophy in Alzheimer’s Disease (AD) and non-pathological aging. WMH, a marker of cerebral small-vessel disease, was measured from fluid-attenuated inversion recovery (FLAIR) MRI scans using the BaMoS method. Hippocampal, ventricular, and grey matter volumes, as well as WMH, were normalized by the total intracranial volume (TIV) to account for intersubject variations in head size.

Exploratory efficacy outcomes included cognitive decline and cognitive improvement, change in the MoCA score, memory and executive functioning composites evaluated with the whole neuropsychological battery (four memory test scores and six executive functioning test scores), prioritizing maximum diversity in its composition. Accordingly, the memory composite included: (i) the FCSRT TR; (ii) the FCSRT-DFR (iii) the LM-IR; and (iv) the LM-DR (history C). The executive functions composite included: (i) the Coding test score; (ii) the Stroop Interference score; (iii) the FDT-inhibition score; (iv) the FDT-Flexibility score; (v) the Animal Fluency test; and (vi) the Digit Span Backwards test. We defined cognitive decline and cognitive improvement according to reliable change indexes from standardized regression-based formulas (RCI_SRB_)^19,20^. First, ‘*T* scores’ of global cognition (measured with the primary outcome PACC-exe), memory and executive functions composites (exploratory outcomes) after 12 months (T_1_) were estimated using linear regression models with baseline scores (T_0_) as predictors, using the formula T_1_’=*b*T_0_+*c*, with T_1_’ indicating the predicted T_1_ score_,_ *b* representing the regression slope and *c* the regression intercept. Then, RCI_SRB_ were calculated as RCI_SRB_= (T_1_- T_1_’)/SEE, where SEE is the standard error of the estimate of the regression equation. A score of ±1.645 is typically considered a ‘reliable change’ as this cut-off indicates that 90% of change scores will fall within this range in a normal distribution. Cognitive decline (i.e. non-respondents) included participants experiencing a reliable decline in cognition after 12 months (RCI_SRB_ ≤ -1.645). Cognitive improvement (i.e. respondents) included participants experiencing a reliable improvement in cognition after 12 months (RCI_SRB_ ≥ 1.645). These analyses were restricted to the MI groups.

## MCI diagnosis

MCI diagnosis was based on evidence of modest cognitive decline (a score below 1.5 standard deviations in the age and education-matched reference group, Neuronorma Scale 5 or below, or percentile 7 or below) from the subject's baseline level of performance as evidenced by decline in any of the following domains: (a) memory (decline in the two main variables of the FCSRT: total immediate recall and total delayed recall), (b) executive functioning (decline in at least 3 of the 4 main results of the FDT-Flexibility, WAIS-IV total Digits, Stroop-Interference, WAIS-IV Digit Span subtest (total score) and WAIS-IV Coding Test), and (c) language (decline in both Semantic Verbal Fluency test and Boston Naming test). Additionally, decline in one of the main memory variables plus decline of two variables from other domains (executive functioning and/or language) could be also reasons for MCI diagnosis. Clinical judgment, considering semiology and pathological behavioral/ performance aspects, also justified an impairment for MCI.

## Safety parameters definitions

Recorded adverse events (AEs) and serious adverse events (SAEs) were monitored and treated according to local standards of care, and were followed until recovery. The severity (intensity) of AEs and SAEs was rated as mild (discomfort noticed, but no disruption of normal daily activity), moderate (discomfort sufficient to reduce or affect normal daily activity), or severe (incapacitating with inability to work or to perform normal daily activity).

According to the ICH guideline for Good Clinical Practice, AEs included (i) any unfavorable and unintended sign (including an abnormal laboratory finding), symptom, or disease temporally associated with the investigational product (it means, along the treatment period), whether or not considered related to the investigational product; (ii) any new disease or exacerbation of an existing disease (i.e., a worsening in the frequency or severity of a known condition); (iii) recurrence of an intermittent medical condition (e.g. headache) not present at baseline; and (iv) any deterioration in a laboratory value or other clinical test, that is associated with symptoms or leads to a change in study treatment or concomitant treatment.

SAEs included those adverse events that met the following criteria: (i) fatal; (ii) life threatening (*i.e.* the adverse event, in the view of the investigator, places the participant at immediate risk of death); (iii) requires or prolongs inpatient hospitalization; (iv) results in persistent or significant disability/incapacity (*i.e.* the adverse event results in substantial disruption of the participants’ ability to conduct normal life functions); (v) significant medical event in the investigator's judgment (e.g. may jeopardize the participant or may require medical/surgical intervention to prevent one of the outcomes listed above); (vi) the occurrence of a clinical seizure should be reported as a SAE if it requires treatment, hospitalization, does not resolve spontaneously or last longer than 3 minutes; and (vii) the occurrence of QTcF value above 500 msec. or a change from baseline by more than 60 msec., if confirmed in a repeated EGC to be recorded within 30 minutes.

AEs and SAEs are reported as subject counts, event counts, and frequencies per study group.

## Baseline characteristics

Baseline type 2 diabetes was defined by previous clinical diagnosis of diabetes, or use of anti-diabetic medication, or use of insulin, or HbA1c ≥ 6.5%, or fasting plasma glucose ≥ 126 mg/dL. Metabolic syndrome^21^ was defined as the presence of at least three of the following criteria: hypertriglyceridemia (triglycerides ≥ 150 mg/dL or use of lipid modifying agents), hyperglycemia (fasting plasma glucose ≥ 100 mg/dL or use of anti-diabetic medication), hypertension (systolic blood pressure ≥ 130 mmHg and diastolic blood pressure ≥ 85 mmHg), reduced HDL-c (< 50 mg/dL in females and < 40 mg/dL in males), and abdominal obesity (waist circumference ≥ 80 in females and ≥ 94 in males). The risk of obstructive sleep apnea syndrome was assessed with the Berlin Questionnaire^22^, which evaluates the presence and frequency of snoring behavior, wake time sleepiness or fatigue, and a history of obesity and/or hypertension. Cognitive reserve was evaluated with the Cognitive Reserve Questionnaire (CRQ)^23^, that comprises eight questions about education, employment, languages, musical education, reading habits and use of intellectual games (e.g. chess, puzzles), whose total score serves as a proxy for cognitive reserve. The Patients Activation Assessment (PAM-13) was used to assess a person’s underlying knowledge, skills and confidence integral to managing his or her own health and healthcare^24^.

## Magnetic Resonance Imaging (MRI)

The MRI acquisition protocol was implemented in a 3T Philips Ingenia CX scanner with a 32-channel head coil and included a high-resolution T1-weighted 3D-TFE sequence (voxel size = 0.75 mm isotropic, flip angle = 8º, repetition time = 9.9 ms, echo time = 4.6 ms, TI = 900 ms), a T2-weighted sequence acquired using a turbo spin echo pulse sequence (TR/TE = 2500/264 ms, flip angle = 90°, FOV = 180 x 250 x 250 mm3, sagittal orientation, voxel size = 0.5 x 0.98 x 0.98 mm3), and a fluid-attenuated inversion recovery (FLAIR) sequence (voxel size = 1 mm isotropic, flip angle = 90º, repetition time = 5000 ms, echo time = 312 ms, TI = 1700 ms).

Freesurfer 7.1 was used to parcellate the brain, including hippocampal subfields and composite measurements of cortical thickness in brain regions known to undergo cortical thinning in AD and aging were calculated. The method used to compute the AD and aging signature composites has been published as described previously. ^25,26^. White Matter Hyperintensities (WMH) were segmented from T1-weighted, T2-weighted and FLAIR scans with the Bayesian Model Selection (BaMoS), a hierarchical fully-unsupervised model selection framework^27^. Regional values of WMH were obtained by averaging lesions within regions considering lobar boundaries (frontal, parietal, temporal, and occipital) and distance between the ventricular surface and cortex (4 layers).

## Blood AD biomarkers

Plasma Aβ42/40, GFAP, and NfL were measured with the Quanterix Neurology 4-Plex E Advantage Kit (#103670), while plasma p-tau181 was measured with the Quanterix p-tau181 immunoassay (#103714, Simoa® P-tau-181 V2 Advantage Kit). All Simoa assays were performed on the Simoa HD-X (Quanterix, Billerica, MA, USA) at the Barcelonaβeta Brain Research Center.

## Dementia risk scores and cardiovascular risk factors

The CAIDE score included seven factors: age, sex, education, hypertension, obesity, hypercholesterolemia, and physical activity. The LIBRA index included 12 factors: coronary heart disease, chronic kidney disease, hypertension, hypercholesterolemia, diabetes, depression, high cognitive activity, obesity, low-to-moderate alcohol use, physical inactivity, smoking and healthy diet (**Table S6**). Visceral fat index (ranges 1 to 59; values ≤ 12 indicate healthy level of visceral fat, whereas values >12 indicate excess levels), muscular and fat mass (in kg, used to calculate the ratio muscular-to-fat mass) were obtained using Advanced Dual Bioelectrical Impedance Analysis Technology (BIA) in a Tanita® scale. Insulin resistance was estimated using the homeostasis model assessment of insulin resistance (HOMA-IR) index with the following formula: ((fasting insulin in µUI/mL) × (fasting glucose in mg/dL))/405)^28^. Beta-cell function (HOMA-B index) was calculated with the following formula (((fasting insulin in µUI/mL) × 20) / (fasting glucose in mg/dL - 3.5))^28^. The triglycerides-glucose index (TyG) was calculated as ((Ln(triglycerides in mg/dL × fasting glucose in mg/dL))/2) and it is expressed in a logarithmic scale^29^.

**Table S6. Operationalization of the LIBRA factors in the PENSA Study.**

| **Factor** | **Operationalization** | **Weight** |
| --- | --- | --- |
| Coronary Heart Disease | Based on two questions from the medical history questionnaire at the baseline visit: history of angina pectoris or history myocardial infarction | +1.0 |
| Chronic Kidney Disease | Presence of estimated glomerular filtration rate (eGFR) value < 60 | +1.1 |
| Hypertension | Systolic blood pressure > 140 mmHg or history of hypertension | +1.6 |
| Hypercholesterolemia | Total cholesterol level > 240 mg/dL | +1.4 |
| Diabetes | Diabetes as indicated on the medical history questionnaire at the baseline visit | +1.3 |
| Depression | A score of ≥ 7 points on the Hospital Anxiety and Depression Scale (HADS) depression score. | +2.1 |
| High Cognitive Activity | Belonging to the 3^rd^ tercile of the Cognitive Reserve Questionnaire. | -3.2 |
| Obesity | Body mass index (BMI) ≥ 30 kg/m^2^ | +1.6 |
| Low/moderate alcohol use | Non-abstinent but consumption of alcohol of ≤10g/week in women and ≤ 20g/week in men | -1.0 |
| Physical inactivity | Reporting < 3,000 metabolic equivalent tasks (METs)-min/week in the Spanish Short Version of the Minnesota Leisure Time Physical Activity Questionnaire | +1.1 |
| Smoking | Current smokers as indicated on the medical history questionnaire at the baseline diet | +1.5 |
| Healthy diet | Adherence to the Mediterranean diet (MedDiet) as measured with a score of ≥ 10 points in the MEDAS-14 questionnaire | -1.7 |
| Total score | Sum of the individual LIBRA weights | -5.9 to 12.7 |

## Olfactory functioning, lifestyle and physical functioning measures

Olfactory functioning was assessed with the University of Pennsylvania Smell Identification Test (UPSIT)^30^. Adherence to the MedDiet was assessed with the 14-point Mediterranean Diet Adherence Screener (MEDAS-14)^31^. A MEDAS-14 score ≥ 10 points was considered a high MedDiet adherence. Physical activity levels measured as metabolic equivalent tasks -METs-minute/week were assessed with the Spanish Short Version of the Minnesota Leisure Time Physical Activity Questionnaire (VREM)^32^. Participants were categorized as sedentary (<625 MET-min/week), moderately active (625-1500 MET-min/week), active (1500-2500 MET-min/week) and very active (≥2500 MET-min/week). The UPSIT, MEDAS-14 and VREM were administered in an interview format delivered by trained professionals. The Grip Strength Test and the 5 time sit-to-stand test (in seconds) of the Short Physical Performance Battery (SPPB)^33^ were used to evaluate the upper body and lower body muscle strength, respectively. In the Grip Strength Test, the maximum weight achieved with the dominant hand after three trials with each arm is reported in kg. In participants allocated to the multimodal intervention groups, physical fitness was additionally evaluated with the Senior Fitness Test (SFT)^34^. The SFT is a battery of physical tests designed for adults aged over 60 years to evaluate strength, endurance, balance, agility, and flexibility. It includes the following seven tests: (i) *30-Second* *Chair Stand Test*: requires repeated standing and sitting in a chair for 30 seconds. The number of stands or repetitions is recorded. This exercise gathers lower body strength. (ii) *30 -Second Arm Curl Test*: individuals might lift 2 kg weight repeatedly (for females) and 3 kg weight (for males) for 30 seconds. Lifted repetitions are count. It measures upper body strength. (iii) 6*-Minute Walk Test*: individuals walk around a 45.72 m rectangular circuit divided into 4.57m segments. The laps done in six minutes are counted considering the segments done, as if it is not an exact lap, and multiplied by the total meters. It reflects aerobic endurance. (iv) *Chair Sit-and-Reach* *Test*: subjects sit on a bench or chair with one leg stretched out with the foot facing the ceiling and try to reach the tip of their toes to the tip of their foot. It reflects lower body flexibility while measuring it in cm. (v) *Back Scratch Test*: using one hand above the shoulder and one below, subjects must try to touch the fingers of both hands behind their back. It is measured in cm and reflects upper body flexibility. (vi) 8-*Foot Up-and-Go Test*: subjects must be seated in a chair, which has in a straight line and at 2.45 m a cone or a mark. They should stand up and turn around the cone and sit down again. The measurement is the time (seconds) between standing up and sitting down again, it measures agility and dynamic balance. (vii) *2-Minute Step Test*: subjects have to lift one leg by bending the knee 90º, then lower it and lift the other leg in the same way. This is considered one repetition. The measure is the total number of repetitions achieved in 2 minutes, used to evaluate the endurance.

## Functionality measures

Self-reported questionnaires by study participants or relatives were completed using the LimeSurvey open-source survey software.

Measures of adaptive behavior were obtained with the Adaptive Behavior Assessment System – Second Edition (ABAS-II)^35^ for adults (ages 16 to 89), which includes 239 items that assess the individual’s competence (in terms of behavior frequency) in 10 different skill areas: (i) *communication abilities* (i.e. to talk, listen, engage in conversation and provide a response), (ii) *community use* (i.e. use of community resources such as shopping or getting around the neighborhood), (iii) *functional academics* (i.e. skills related to reading, writing, mathematics and other areas necessary for independent daily functioning), (iv) *home living* (i.e. home care skills such as tidying, cleaning, repairing, and caring for objects), (v) *health and safety* (i.e. skills related to maintaining an adequate state of health, such as respecting safety rules, using medicines and showing caution), (vi) *leisure* (i.e. participation in recreational activities, compliance with the rules of the games, and leisure planning), (vii) *self‐care* (i.e. activities related to food, clothing and hygiene), (viii) *self‐direction* (i.e. performing tasks, complying with deadlines and time constraints, following instructions and other activities involving responsibility and self-monitoring), (ix) *social interaction* (i.e. interacting socially, getting along with others, making friends and maintaining friendships, showing good manners and communicating one's emotions) and (x) *working/labor skills* (functional skills to perform successfully on the job, including performing assigned tasks and complying with schedules and instructions). Scalar scores for each domain were computed with a mean of 10 and a standard deviation of 3. These scores were used to obtain a General Adaptive Composite. All answers to this questionnaire were reported by an informant. Those items rated as ‘guessed’ by the informant were scored as zero, in order to avoid subjective judgments concerning functional changes. Higher scores indicate higher adaptive skills and independency in everyday living. Given that most individuals in our sample were already retired, scores in the work skill area were not included in the analyses and were not considered for calculating the General Adaptive Composite.

Quality of life was assessed with the Spanish version of the EuroQol 5 dimensions 5 Levels (EQ-5D-5L)^36,37^ questionnaire, and the Spanish version of the World Health Organization quality of life questionnaire^38^. The EQ-5D-5L is a brief, multi-attribute, generic, health status measure composed of 5 questions with Likert response options (descriptive system) and a visual analogue scale (EQ-VAS). The latter asks patients to rate their own health from 0 to 100 (the worst and best imaginable health, respectively). The descriptive system covers 5 dimensions of health (mobility, self-care, usual activities, pain or discomfort, and anxiety or depression) with 5 levels of severity in each dimension (no problems, slight problems, moderate problems, severe problems, and unable to perform or extreme problems). The WHOQOL-BREF comprises 26 items, categorized into four domains: physical health (7 items), psychological well-being (6 items), social connections (3 items), and environmental health (8 items). Additionally, it includes items addressing overall quality of life (QOL) and general health. Each item in the WHOQOL-BREF is assessed on a five-point ordinal scale. These scores are then linearly transformed to a 0–100 scale. The physical health domain encompasses aspects such as mobility, daily activities, functional capacity, energy, pain, and sleep. Within the psychological domain, assessments cover self-image, negative thoughts, positive attitudes, self-esteem, mentality, learning ability, memory concentration, religion, and mental status. The social relationships domain explores personal relationships, social support, and aspects of one's sex life. Lastly, the environmental health domain addresses issues such as financial resources, safety, access to health and social services, living conditions, opportunities for skill development, recreation, general environment (including noise and air pollution), and transportation.

Quality of sleep was assessed with the Pittsburg Sleep Quality Index (PSQI)^39^ and the Epworth sleepiness scale^40^. Psychological distress was assessed with the General Health Questionnaire (GHQ-28)^41^, and the Hospital Anxiety and Depression Scale (HADS)^42^.

## Adherence to the multimodal intervention

Participation in intervention activities was manually registered by study staff. The maximum number of offered intervention activities during the intervention period was 9 individual nutrition counselling visits, 1-2 gymnasium classes/week (equivalent to a total of 30 to 46 sessions depending on the study sub-group), 10 psychoeducation group sessions, 10 social stimulation activities and 12 cognitive training sessions/month (equivalent to a total of 144 sessions). Intervention participation was the mean proportion of the completed sessions. Compliance with daily EMAs was the average weekly adherence, calculated as the number of days that participants completed the dietary EMAs questionnaire divided by 7 days. Compliance with the Fitbit activity tracker was assessed with the number of complete observations, defined as at least 600 minutes of valid minute heart rate signal per day (i.e., wear time indicator).

## Online-Only References

1 Boronat A, Clivillé J, Soldevila-Domenech N, *et al.* Mobile Device-assisted Dietary Ecological Momentary Assessments for the Evaluation of the Adherence to the Mediterranean Diet in a Continuous Manner. *JoVE* 2021; **175**: 1–17.

2 Tudor-Locke C, Craig CL, Aoyagi Y, *et al.* How many steps/day are enough? For older adults and special populations. *Int J Behav Nutr Phys Act* 2011; **8**: 80.

3 Bassett DR, Toth LP, LaMunion SR, Crouter SE. Step Counting: A Review of Measurement Considerations and Health-Related Applications. Sport. Med. 2017; **47**: 1303–15.

4 Nelson ME, Rejeski WJ, Blair SN, *et al.* Physical activity and public health in older adults: Recommendation from the American College of Sports Medicine and the American Heart Association. *Med Sci Sports Exerc* 2007; **39**: 1435–45.

5 Fitbit. Help article: What are active minutes? 2017.

6 Borg G. Perceived exertion as an indicator of somatic stress. *Scand J Rehabil Med* 1970; **2**: 92–8.

7 Soldevila-Domenech N, De Toma I, Forcano L, *et al.* Intensive assessment of executive functions derived from performance in cognitive training games. *iScience* 2023; **26**: 1–26.

8 Buschke H. Cued recall in amnesia. *J Clin Neuropsychol* 1984; **6**: 433–40.

9 Wechsler D. Wechsler Memory Scale IV (WMS-IV). New York, NY: Psychological Corporation, 2009.

10 Wechsler D. Manual for the Wechsler Adult Intelligence Scale, Revised. New York: Psychological Corporation, 1981.

11 Stroop JR. Studies of interference in serial verbal reactions. *J Exp Psychol* 1935; **18**: 643–62.

12 Chafetz MD, Matthews LH. A New interference score for the Stroop test. *Arch Clin Neuropsychol* 2004; **19**: 555–67.

13 Jardim De Paula J, Teixeira De Ávila R, De Souza Costa D, *et al.* Assessing processing speed and executive functions in low educated older adults: the use of the Five Digits Test in patients with Alzheimer’s Disease, Mild Cognitive Impairment and Major Depressive Disorder. *Clin Neuropsychiatry* 2011; **8**: 339–46.

14 Benton A, Hamsher K, Sivan AB. Multilingual Aphasia Examination: Manual of instruction. Iowa City: AJA Associates, 1994.

15 Wechsler D. Wechsler Adult Intelligence Scale—Fourth Edition Administration and Scoring Manual. San Antonio, TX: Pearson, 2008.

16 Folstein MF, Folstein SE, McHugh PR. ‘Mini-mental state’. A practical method for grading the cognitive state of patients for the clinician. *J Psychiatr Res* 1975; **12**: 189–98.

17 Ojeda N, Del Pino R, Ibarretxe-Bilbao N, Schretlen DJ, Pena J. Montreal Cognitive Assessment Test: normalization and standardization for Spanish population. *Rev Neurol* 2016; **63**: 488–96.

18 Casals-Coll M, Sánchez-Benavides G, Meza-Cavazos S, *et al.* Spanish multicenter normative studies (NEURONORMA project): Normative data and equivalence of four BNT short-form versions. *Arch Clin Neuropsychol* 2014; **29**: 60–74.

19 McSweeny AJ, Naugle RI, Chelune GJ, Luders H. ‘T Scores for Change’: An illustration of a regression approach to depicting change in clinical neuropsychology. *Clin Neuropsychol* 1993; **7**: 300–12.

20 Duff K. Evidence-based indicators of neuropsychological change in the individual patient: Relevant concepts and methods. *Arch Clin Neuropsychol* 2012; **27**: 248–61.

21 Alberti KGMM, Eckel RH, Grundy SM, *et al.* Harmonizing the metabolic syndrome: A joint interim statement of the international diabetes federation task force on epidemiology and prevention; National heart, lung, and blood institute; American heart association; World heart federation; International. *Circulation* 2009; **120**: 1640–5.

22 Netzer N, Stoohs R, Netzer C, Clark K, Strohl K. Using the Berlin Questionnaire To Identify Patients at Risk for the Sleep Apnea Syndrome. *Ann Intern Med* 1999; **131**: 485–91.

23 Rami L, Valls-Pedret C, Bartrés-Faz D, *et al.* Cognitive reserve questionnaire. Scores obtained in a healthy elderly population and in one with Alzheimer’s disease. *Rev Neurol* 2011; **52**: 195–201.

24 Moreno-Chico C, González-de Paz L, Monforte-Royo C, Arrighi E, Navarro-Rubio MD, Fernández-Puebla AG. Adaptation to European Spanish and psychometric properties of the Patient Activation Measure 13 in patients with chronic diseases. *Fam Pract* 2017; **34**: 627–34.

25 Dickerson BC, Stoub TR, Shah RC, *et al.* Alzheimer-signature MRI biomarker predicts AD dementia in cognitively normal adults. *Neurology* 2011; **76**: 1395–402.

26 Schwarz CG, Gunter JL, Wiste HJ, *et al.* A large-scale comparison of cortical thickness and volume methods for measuring Alzheimer’s disease severity. *NeuroImage Clin* 2016; **11**: 802–12.

27 Sudre CH, Cardoso MJ, Bouvy WH, Biessels GJ, Barnes J, Ourselin S. Bayesian Model Selection for Pathological Neuroimaging Data Applied to White Matter Lesion Segmentation. *IEEE Trans Med Imaging* 2015; **34**: 2079–102.

28 Matthews DR, Hosker JP, Rudenski AS, Naylor BA, Treacher DF, Turner RC. Homeostasis model assessment: insulin resistance and β-cell function from fasting plasma glucose and insulin concentrations in man. *Diabetologia* 1985; **28**: 412–9.

29 Simental-Medía L, Rodríguez-Moran M, Guerrero-Romero F. The Product of Fasting Glucose and Triglycerides As Surrogate for Identifying Insulin Resistance in Apparently Healthy Subjects. *Metab Syndr Relat Disord* 2008; **6**: 1–6.

30 Doty RL, Frye RE, Agrawal U. Internal consistency reliability of the fractionated and whole University of Pennsylvania Smell Identification Test. *Percept Psychophys* 1989; **45**: 381–4.

31 Schröder H, Zomeño MD, Martínez-González MA, *et al.* Validity of the energy-restricted Mediterranean Diet Adherence Screener. *Clin Nutr* 2021; **40**: 4971–9.

32 Comellas AR, Pera G, Diez JMB, *et al.* Validation of a spanish short version of the minnesota leisure time physical activity questionnaire (vrem). *Rev Esp Salud Publica* 2012; **86**: 495–508.

33 Guralnik J, Simonsick E, Ferrucci L, *et al.* A short physical performance battery assessing lower extremity function: association with self-reported disability and prediction of mortality and nursing home admission. *J Gerontol* 1994; **49**: M85-94.

34 Rikli RE, Jones CJ. Development and validation of criterion-referenced clinically relevant fitness standards for maintaining physical independence in later years. *Gerontologist* 2013; **53**: 255–67.

35 Oakland T, Harrison P. ABAS-II. Clinical use and interpretation. London: Springer, New York, NY, 2008 DOI:https://doi.org/10.1016/B978-0-12-373586-7.X0001-X.

36 Garcia-Gordillo MA, Adsuar JC, Olivares PR. Normative values of EQ-5D-5L: in a Spanish representative population sample from Spanish Health Survey, 2011. *Qual Life Res* 2016; **25**: 1313–21.

37 Hernandez G, Garin O, Pardo Y, *et al.* Validity of the EQ–5D–5L and reference norms for the Spanish population. *Qual Life Res* 2018; **27**: 2337–48.

38 Skevington SM, Lotfy M, O’Connell KA. The World Health Organization’s WHOQOL-BREF quality of life assessment: Psychometric properties and results of the international field trial a Report from the WHOQOL Group. *Qual Life Res* 2004; **13**: 299–310.

39 Royuela Rico A, Macías Fernández JA. Propiedades clinimétricas de la versión castellana del Cuestionario de Pittsburgh. *Vigilia-Sueño* 1997; **9**: 81–94.

40 Chiner E, Arriero JM, Signes-Costa J, Marco J, Fuentes I. Validación de la versión española del test de somnolencia Epworth en pacientes con síndrome de apnea de sueño. *Arch Bronconeumol* 1999; **35**: 422–7.

41 Goldberg DP, Gater R, Sartorius N, *et al.* The validity of two versions of the GHQ in the WHO study of mental illness in general health care. *Psychol Med* 1997; **27**: 191–7.

42 Zigmond AS, Snaith RP. The Hospital Anxiety and Depression Scale. *Acta Psychiatr Scand* 1983; **67**: 361–70.

# Appendix 2. Supplementary Results

# Supplementary Tables

### Table 1. SCD characteristics of all included participants.

|  | **MLI+EGCG (n=52)** | **MLI+Placebo**  **(n=52)** | **NRG (n=25)** |
| --- | --- | --- | --- |
| ***Self-reported SCD status:*** |  |  |  |
| **Do you perceive memory or cognitive difficulties?** | 52 (100) | 52 (100) | 25 (100) |
| **Have you consulted a doctor?** |  |  |  |
| Has consulted a doctor | 26 (50.0) | 25 (48.1) | 14 (56.0) |
| Wants to consult a doctor | 18 (34.6) | 18 (34.6) | 9 (36.0) |
| No | 8 (15.4) | 9 (17.3) | 2 (8.0) |
| **Are you worried about the decline?** | 39 (75.0) | 43 (82.7) | 22 (88.0) |
| **Duration** |  |  |  |
| <2 years | 14 (26.9) | 11 (21.2) | 7 (28.0) |
| Between 2-5 years | 24 (46.2) | 21 (40.4) | 10 (40.0) |
| >5 years | 14 (26.9) | 20 (38.5) | 8 (32.0) |
| **Is memory affected?** | 51 (98.1) | 51 (98.1) | 24 (96.0) |
| **Is concentration affected?** | 36 (69.2) | 30 (57.7) | 19 (76.0) |
| **Is language affected?** | 35 (67.3) | 40 (76.9) | 18 (72.0) |
| **Is visuospatial ability affected?** | 8 (15.4) | 3 (5.8) | 2 (8.0) |
| **Which is the main domain affected?** |  |  |  |
| Memory | 30 (68.2) | 30 (62.5) | 12 (54.5) |
| Concentration/ Attention | 3 (6.8) | 5 (10.4) | 5 (22.7) |
| Language | 10 (22.7) | 13 (27.1) | 5 (22.7) |
| Other | 1 (2.3) | 0 (0.0) | 0 (0.0) |
| **Lower cognition than people of your same age?** | 14 (26.9) | 10 (19.2) | 9 (36.0) |
| Have your relatives noticed the decline? |  |  |  |
| Yes | 23 (44.2) | 20 (38.5) | 10 (40.0) |
| No | 26 (50.0) | 28 (53.8) | 12 (48.0) |
| Do not know | 3 (5.8) | 4 (7.7) | 3 (12.0) |
| **Are your relatives worried about the decline?** |  |  |  |
| Yes | 9 (39.1) | 10 (50.0) | 5 (50.0) |
| No | 12 (52.2) | 9 (45.0) | 5 (50.0) |
| Do not know | 2 (8.7) | 1 (5.0) | 0 (0.0) |
| ***SCD status reported by a relative**** |  |  |  |
| **Have you noticed the decline?** | 27 (73.0) | 26 (60.5) | 9 (60.0) |
| Questions administered by experienced neurologist or neuropsychologists.  *n=98 participants with information available. NRG= Non-randomized group. EGCG= epigallocatechin-3-gallate. MLI= multimodal intervention. SCD= subjective cognitive decline.  *Values are n (%).* | | | |

### Table 2. Self-reported adverse effects (as ‘System Organ Class Terms’) by treatment group during the 15 months of follow-up in included participants.

|  | **NRG (N=25)** | | **MLI+EGCG (N=52)** | | | **MLI+placebo (N=52)** | | |
| --- | --- | --- | --- | --- | --- | --- | --- | --- |
|  | **N events** | **Subjects  N (%)** | **N events** | **Subjects  N (%)** | | **N events** | **Subjects  N (%)** | |
| ***Serious adverse events*** | | | | | | | | |
| All | 7 | 3 (12.0) | 6 | | 5 (9.6) | 1 | | 1 (2.0%) |
| Cardiac disorders | 1 | 1 (4.0) | 0 | |  | 0 | |  |
| Hepatobiliary disorders | 1 | 1 (4.0) | 1 | | 1 (1.9) | 0 | |  |
| Infections and infestations | 1 | 1 (4.0) | 1 | | 1 (1.9) | 0 | |  |
| Injury, poisoning and procedural complications | 0 |  | 1 | | 1 (1.9) | 0 | |  |
| Investigations | 1 | 1 (4.0) | 0 | |  | 0 | |  |
| Neoplasms benign, malignant and unspecified (incl. cysts and polyps) | 1 | 1 (4.0) | 2 | | 2 (3.8) | 1 | | 1 (2.0) |
| Nervous system disorders | 0 |  | 1 | | 1 (1.9) | 0 | |  |
| Renal and urinary disorders | 1 | 1 (4.0) | 0 | |  | 0 | |  |
| Surgical and medical procedures | 1 | 1 (4.0) | 0 | |  | 0 | |  |
| ***Adverse events*** | | | | | | | | |
| All | 30 | 16 (64.0) | 63 | | 36 (69.2) | 58 | | 40 (76.9) |
| Blood and lymphatic system disorders | 10 | 10 (40.0) | 6 | | 6 (11.5) | 8 | | 8 (15.7) |
| Cardiac disorders | 1 | 1 (4.0) | 3 | | 3 (5.8) | 1 | | 1 (2.0) |
| Endocrine disorders | 0 |  | 3 | | 3 (5.8) | 5 | | 5 (9.8) |
| Gastrointestinal disorders | 0 |  | 1 | | 1 (1.9) | 2 | | 2 (3.9) |
| General disorders and administration site conditions | 0 |  | 1 | | 1 (1.9) | 0 | |  |
| Hepatobiliary disorders | 4 | 3 (12.0) | 7 | | 7 (13.5) | 4 | | 4 (7.8) |
| Infections and infestations | 7 | 6 (24.0) | 13 | | 13 (25.0) | 12 | | 11 (21.6) |
| Injury, poisoning and procedural complications | 1 | 1 (4.0) | 1 | | 1 (1.9) | 2 | | 2 (3.9) |
| Investigations | 0 |  | 1 | | 1 (1.9) | 2 | | 2 (3.9) |
| Metabolism and nutrition disorders | 3 | 3 (12.0) | 13 | | 12 (23.1) | 8 | | 8 (15.7) |
| Musculoskeletal and connective tissue disorders | 0 |  | 1 | | 1 (1.9) | 4 | | 4 (7.8) |
| Nervous system disorders | 1 | 1 (4.0) | 2 | | 2 (3.8) | 2 | | 2 (3.9) |
| Renal and urinary disorders | 2 | 1 (4.0) | 5 | | 5 (9.6) | 5 | | 5 (9.8) |
| Respiratory, thoracic and mediastinal disorders | 0 |  | 1 | | 1 (1.9) | 0 | |  |
| Surgical and medical procedures | 0 |  | 4 | | 3 (5.8) | 3 | | 3 (5.9) |
| Vascular disorders | 1 | 1 (4.0) | 1 | | 1 (1.9) | 0 | |  |
| NRG= Non-randomized group. EGCG= epigallocatechin-3-gallate. MLI= multimodal intervention. | | | | | | | | |

### Table 3. Self-reported adverse effects (as ‘Preferred Terms’) by treatment group during the 15 months of follow-up in included participants.

|  | **NRG (N=25)** | | **MLI+EGCG (N=52)** | | **MLI+placebo (N=52)** | |
| --- | --- | --- | --- | --- | --- | --- |
|  | **N events** | **Subjects  N (%)** | **N events** | **Subjects  N (%)** | **N events** | **Subjects  N (%)** |
| ***Serious adverse events*** |  |  |  |  |  |  |
| All | 7 | 3 (12.0) | 6 | 5 (9.6) | 1 | 1 (2.0) |
| Azotemia | 1 | 1 (4.0) | 0 | 0 (0.0) | 0 | 0 (0.0) |
| Breast neoplasm | 0 | 0 (0.0) | 2 | 2 (3.8) | 0 | 0 (0.0) |
| Cardiac failure | 1 | 1 (4.0) | 0 | 0 (0.0) | 0 | 0 (0.0) |
| COVID-19 | 1 | 1 (4.0) | 0 | 0 (0.0) | 0 | 0 (0.0) |
| Femur fracture | 0 | 0 (0.0) | 1 | 1 (1.9) | 0 | 0 (0.0) |
| Hemangioma | 1 | 1 (4.0) | 0 | 0 (0.0) | 0 | 0 (0.0) |
| Hyperbilirubinemia | 1 | 1 (4.0) | 0 | 0 (0.0) | 0 | 0 (0.0) |
| Hypertransaminasemia | 0 | 0 (0.0) | 1 | 1 (1.9) | 0 | 0 (0.0) |
| Low density lipoprotein increased | 1 | 1 (4.0) | 0 | 0 (0.0) | 0 | 0 (0.0) |
| Pneumonia | 0 | 0 (0.0) | 1 | 1 (1.9) | 0 | 0 (0.0) |
| Spinal laminectomy | 1 | 1 (4.0) | 0 | 0 (0.0) | 0 | 0 (0.0) |
| Transient ischemic attack | 0 | 0 (0.0) | 1 | 1 (1.9) | 0 | 0 (0.0) |
| Transitional cell carcinoma | 0 | 0 (0.0) | 0 | 0 (0.0) | 1 | 1 (2.0) |
| ***Adverse events*** |  |  |  |  |  |  |
| All | 30 | 16 (64.0) | 63 | 36 (69.2) | 58 | 40 (76.9) |
| Abdominal distension | 0 | 0 (0.0) | 1 | 1 (1.9) | 0 | 0 (0.0) |
| Abdominal operation | 0 | 0 (0.0) | 1 | 1 (1.9) | 0 | 0 (0.0) |
| Anemia macrocytic | 1 | 1 (4.0) | 0 | 0 (0.0) | 0 | 0 (0.0) |
| Arachnoid cyst | 1 | 1 (4.0) | 0 | 0 (0.0) | 0 | 0 (0.0) |
| Arteriosclerosis | 0 | 0 (0.0) | 1 | 1 (1.9) | 0 | 0 (0.0) |
| Arthralgia | 0 | 0 (0.0) | 0 | 0 (0.0) | 1 | 1 (2.0) |
| Atrial flutter | 0 | 0 (0.0) | 1 | 1 (1.9) | 0 | 0 (0.0) |
| Bicytopenia | 1 | 1 (4.0) | 0 | 0 (0.0) | 0 | 0 (0.0) |
| Bundle branch block left | 0 | 0 (0.0) | 1 | 1 (1.9) | 0 | 0 (0.0) |
| Cataract operation | 0 | 0 (0.0) | 3 | 2 (3.8) | 0 | 0 (0.0) |
| Cognitive disorder | 0 | 0 (0.0) | 1 | 1 (1.9) | 0 | 0 (0.0) |
| COVID-19 | 5 | 5 (20.0) | 11 | 11 (21.2) | 9 | 9 (17.6) |
| Dental operation | 0 | 0 (0.0) | 0 | 0 (0.0) | 1 | 1 (2.0) |
| Dyslipidemia | 2 | 2 (8.0) | 6 | 6 (11.5) | 5 | 5 (9.8) |
| Epistaxis | 0 | 0 (0.0) | 1 | 1 (1.9) | 0 | 0 (0.0) |
| Fatigue | 0 | 0 (0.0) | 1 | 1 (1.9) | 0 | 0 (0.0) |
| Gamma-glutamyl transferase increased | 0 | 0 (0.0) | 1 | 1 (1.9) | 1 | 1 (2.0) |
| Head injury | 1 | 1 (4.0) | 0 | 0 (0.0) | 0 | 0 (0.0) |
| Hyperbilirubinemia | 4 | 3 (12.0) | 3 | 3 (5.8) | 3 | 3 (5.9) |
| Hypercholesterolemia | 0 | 0 (0.0) | 1 | 1 (1.9) | 1 | 1 (2.0) |
| Hyperchromic anemia | 1 | 1 (4.0) | 0 | 0 (0.0) | 1 | 1 (2.0) |
| Hyperferritinemia | 1 | 1 (4.0) | 0 | 0 (0.0) | 0 | 0 (0.0) |
| Hypertransaminasemia | 0 | 0 (0.0) | 4 | 4 (7.7) | 1 | 1 (2.0) |
| Hypertriglyceridemia | 0 | 0 (0.0) | 3 | 3 (5.8) | 2 | 2 (3.9) |
| Hyperuricemia | 0 | 0 (0.0) | 2 | 2 (3.8) | 0 | 0 (0.0) |
| Hypothyroidism | 0 | 0 (0.0) | 3 | 3 (5.8) | 5 | 5 (9.8) |
| Intracranial aneurysm | 0 | 0 (0.0) | 0 | 0 (0.0) | 1 | 1 (2.0) |
| Iron deficiency anemia | 2 | 2 (8.0) | 0 | 0 (0.0) | 1 | 1 (2.0) |
| Jaw cyst | 0 | 0 (0.0) | 0 | 0 (0.0) | 1 | 1 (2.0) |
| Jaw fracture | 0 | 0 (0.0) | 0 | 0 (0.0) | 1 | 1 (2.0) |
| Lacunar infarction | 0 | 0 (0.0) | 0 | 0 (0.0) | 1 | 1 (2.0) |
| Large intestine polyp | 0 | 0 (0.0) | 0 | 0 (0.0) | 1 | 1 (2.0) |
| Leukocytosis | 0 | 0 (0.0) | 0 | 0 (0.0) | 1 | 1 (2.0) |
| Leukocyturia | 0 | 0 (0.0) | 2 | 2 (3.8) | 0 | 0 (0.0) |
| Leukopenia | 0 | 0 (0.0) | 1 | 1 (1.9) | 0 | 0 (0.0) |
| Lymphopenia | 2 | 2 (8.0) | 0 | 0 (0.0) | 0 | 0 (0.0) |
| Meniscopathy | 0 | 0 (0.0) | 0 | 0 (0.0) | 1 | 1 (2.0) |
| Myocardial ischemia | 0 | 0 (0.0) | 0 | 0 (0.0) | 1 | 1 (2.0) |
| Nephrolithiasis | 1 | 1 (4.0) | 0 | 0 (0.0) | 0 | 0 (0.0) |
| Neutropenia | 1 | 1 (4.0) | 2 | 2 (3.8) | 0 | 0 (0.0) |
| Normochromic normocytic anemia | 1 | 1 (4.0) | 2 | 2 (3.8) | 3 | 3 (5.9) |
| Esophageal spasm | 0 | 0 (0.0) | 0 | 0 (0.0) | 1 | 1 (2.0) |
| Prodromal Alzheimer's disease | 0 | 0 (0.0) | 1 | 1 (1.9) | 0 | 0 (0.0) |
| Prothrombin time prolonged | 0 | 0 (0.0) | 0 | 0 (0.0) | 1 | 1 (2.0) |
| Radius fracture | 0 | 0 (0.0) | 0 | 0 (0.0) | 1 | 1 (2.0) |
| Renal impairment | 1 | 1 (4.0) | 3 | 3 (5.8) | 5 | 5 (9.8) |
| Rhabdomyolysis | 0 | 0 (0.0) | 1 | 1 (1.9) | 0 | 0 (0.0) |
| Rib fracture | 0 | 0 (0.0) | 1 | 1 (1.9) | 0 | 0 (0.0) |
| Rotator cuff syndrome | 0 | 0 (0.0) | 0 | 0 (0.0) | 1 | 1 (2.0) |
| Sebaceous cyst excision | 0 | 0 (0.0) | 0 | 0 (0.0) | 1 | 1 (2.0) |
| Sinus bradycardia | 0 | 0 (0.0) | 1 | 1 (1.9) | 0 | 0 (0.0) |
| Skin infection | 0 | 0 (0.0) | 0 | 0 (0.0) | 1 | 1 (2.0) |
| Stem cell therapy | 0 | 0 (0.0) | 0 | 0 (0.0) | 1 | 1 (2.0) |
| Thrombocytopenia | 1 | 1 (4.0) | 1 | 1 (1.9) | 1 | 1 (2.0) |
| Thrombocytosis | 0 | 0 (0.0) | 0 | 0 (0.0) | 1 | 1 (2.0) |
| Type 2 diabetes mellitus | 0 | 0 (0.0) | 1 | 1 (1.9) | 0 | 0 (0.0) |
| Urinary tract infection | 2 | 2 (8.0) | 2 | 2 (3.8) | 1 | 1 (2.0) |
| Venous thrombosis | 1 | 1 (4.0) | 0 | 0 (0.0) | 0 | 0 (0.0) |
| Ventricular extrasystoles | 1 | 1 (4.0) | 0 | 0 (0.0) | 0 | 0 (0.0) |
| Viral infection | 0 | 0 (0.0) | 0 | 0 (0.0) | 1 | 1 (2.0) |
| NRG= Non-randomized group. EGCG= epigallocatechin gallate. MLI= multimodal intervention. | | | | | | |

### Table 4. Intervention effects on brain structure (MRI) and plasma AD biomarkers in the modified intention-to-treat population.

|  |  | **MLI+EGCG (n=51)** | | **MLI+placebo (n=50)** | | **MLI+EGCG vs. MLI+placebo** | |
| --- | --- | --- | --- | --- | --- | --- | --- |
|  | **Month** | **Mean** | **Mean Change  (95%CI)** | **Mean** | **Mean Change (95%CI)** | **Adjusted mean difference***  **of changes from month 0 (95%CI)** | **p-value** |
| Whole hippocampal volume / TIV (%) | 0 | 0.432 |  | 0.434 |  |  |  |
|  | 12 | 0.424 | -0.008 (-0.011, -0.004) | 0.427 | -0.008 (-0.011, -0.004) | 0.000 (-0.005, 0.005) | 0.985 |
| WMH / TIV (%) | 0 | 0.197 |  | 0.294 |  |  |  |
|  | 12 | 0.203 | 0.013 (-0.002, 0.028) | 0.318 | 0.024 (0.006, 0.042) | -0.004 (-0.028, 0.019) | 0.709 |
| Gray Matter Volume / TIV (%) | 0 | 0.617 |  | 0.599 |  |  |  |
|  | 12 | 0.616 | -0.009 (-0.013, -0.004) | 0.590 | -0.009 (-0.014, -0.004) | 0.003 (-0.004, 0.009) | 0.379 |
| Cortical thickness (mm) | 0 | 2.481 |  | 2.463 |  |  |  |
|  | 12 | 2.473 | -0.015 (-0.030, -0.001) | 2.447 | -0.014 (-0.029, -0.000) | 0.004 (-0.015, 0.023) | 0.662 |
| Cortical thickness AD signature (mm) | 0 | 2.431 |  | 2.422 |  |  |  |
|  | 12 | 2.428 | -0.016 (-0.027, -0.005) | 2.409 | -0.011 (-0.024, 0.002) | -0.002 (-0.018, 0.015) | 0.822 |
| Cortical thickness Aging signature (mm) | 0 | 2.263 |  | 2.258 |  |  |  |
|  | 12 | 2.262 | -0.012 (-0.022, -0.002) | 2.244 | -0.010 (-0.021, 0.001) | 0.000 (-0.015, 0.015) | 0.965 |
| Ventricular volume / TIV (%) | 0 | 1.749 |  | 1.815 |  |  |  |
|  | 12 | 1.805 | 0.099 (0.075, 0.124) | 1.928 | 0.116 (0.087, 0.145) | -0.011 (-0.046, 0.023) | 0.513 |
| Ventricular volume (cm3) | 0 | 25.57 |  | 26.72 |  |  |  |
|  | 12 | 26.47 | 1.47 (1.10, 1.83) | 28.52 | 1.83 (1.37, 2.29) | -0.27 (-0.79, 0.25) | 0.306 |
| Ab40 (pg/mL) | 0 | 113.4 |  | 115.3 |  |  |  |
|  | 12 | 105.2 | -8.4 (-12.6, -4.2) | 110.0 | -5.2 (-10.9, 0.6) | -4.0 (-9.5, 1.5) | 0.149 |
| Ab42 (pg/mL) | 0 | 5.652 |  | 5.573 |  |  |  |
|  | 12 | 5.673 | -0.020 (-0.328, 0.288) | 5.901 | 0.371 (0.069, 0.672) | -0.329 (-0.698, 0.040) | 0.080 |
| Ab42/Ab40 ratio | 0 | 0.05 |  | 0.05 |  |  |  |
|  | 12 | 0.05 | 0.00 (0.00, 0.01) | 0.05 | 0.01 (0.00, 0.01) | -0.001 (-0.004, 0.002) | 0.390 |
| p-tau181 (pg/mL) | 0 | 27.4 |  | 26.2 |  |  |  |
|  | 12 | 24.7 | -1.3 (-4.2, 1.7) | 26.6 | 0.6 (-1.8, 3.0) | -1.9 (-4.9, 1.2) | 0.224 |
| NfL (pg/mL) | 0 | 22.2 |  | 21.0 |  |  |  |
|  | 12 | 24.6 | 2.9 (-0.4, 6.2) | 21.9 | 0.9 (-0.2, 2.0) | 1.9 (-1.5, 5.3) | 0.269 |
| GFAP (pg/mL) | 0 | 122.3 |  | 128.8 |  |  |  |
|  | 12 | 115.6 | 1.7 (-5.3, 8.8) | 123.9 | -4.2 (-12.1, 3.8) | 5.4 (-5.2, 16.0) | 0.312 |
| EGCG= epigallocatechin-3-gallate. MLI= multimodal intervention. AD= Alzheimer’s disease. Aβ = amyloid beta. EGCG= epigallocatechin-3-gallate. GFAP= Glial fibrillary acidic protein. NfL= neurofilament light. TIV= total intracranial volume. WMH= white matter hyperintensities. p-tau-181= phosphorylated tau 181. *Adjusted mean differences (AMD) are obtained from ANCOVA models for differences from baseline adjusted for baseline scores. In the case of positive changes from baseline, a positive value of the AMD indicates larger changes in the MLI+EGCG group. In the case of negative changes from baseline, a negative value of the AMD indicates larger absolute changes in the MLI+EGCG group. | | | | | | | |

### Table 5. Intervention effects on dementia risk scores, anthropometric factors and cardiovascular risk factors in the modified intention-to-treat population.

|  |  | **MLI+EGCG (n=51)** | | **MLI+placebo (n=50)** | | **MLI+EGCG vs. MLI+placebo** | |
| --- | --- | --- | --- | --- | --- | --- | --- |
|  | **Month** | **Mean** | **Mean Change  (95%CI)** | **Mean** | **Mean Change (95%CI)** | **Adjusted mean difference***  **of changes from month 0 (95%CI)** | **p-value** |
| CAIDE | 0 | 7.5 |  | 7.6 |  |  |  |
|  | 12 | 7.1 | -0.3 (-0.6, 0.0) | 7.3 | -0.3 (-0.6, -0.1) | 0.0 (-0.3, 0.4) | 0.881 |
| Weight (kg) | 0 | 68.9 |  | 69.4 |  |  |  |
|  | 12 | 67.6 | -1.0 (-1.7, -0.2) | 68.1 | -1.4 (-2.7, -0.1) | 0.4 (-1.0, 1.7) | 0.610 |
| Waist circumference (cm) | 0 | 94.0 |  | 95.3 |  |  |  |
|  | 12 | 92.2 | -1.0 (-3.4, 1.4) | 93.1 | -2.3 (-3.8, -0.8) | 1.1 (-1.7, 3.9) | 0.450 |
| Diastolic BP (mmHg) | 0 | 69.9 |  | 72.9 |  |  |  |
|  | 12 | 67.5 | -2.2 (-6.8, 2.4) | 68.2 | -5.2 (-8.8, -1.5) | 0.7 (-4.3, 5.7) | 0.779 |
| Systolic BP (mmHg) | 0 | 131.3 |  | 132.3 |  |  |  |
|  | 12 | 117.2 | -12.9 (-18.3, -7.5) | 123.1 | -9.2 (-13.8, -4.6) | -4.8 (-11.3, 1.6) | 0.142 |
| Total cholesterol (mg/dL) | 0 | 204.6 |  | 206.2 |  |  |  |
|  | 12 | 198.9 | -5.8 (-12.9, 1.3) | 198.6 | -7.6 (-15.2, 0.0) | 1.3 (-8.0, 10.6) | 0.782 |
| HDL-c (mg/dL) | 0 | 62.2 |  | 61.7 |  |  |  |
|  | 12 | 61.0 | -1.2 (-3.7, 1.2) | 61.9 | 0.2 (-2.6, 2.9) | -1.3 (-4.8, 2.2) | 0.460 |
| LDL-c (mg/dL) | 0 | 128.8 |  | 130.5 |  |  |  |
|  | 12 | 121.7 | -7.1 (-13.9, -0.3) | 122.3 | -8.2 (-13.9, -2.4) | 0.5 (-7.4, 8.5) | 0.892 |
| HDL-c/Total cholesterol ratio (%) | 0 | 30.6 |  | 30.5 |  |  |  |
|  | 12 | 31.0 | 0.4 (-0.9, 1.7) | 31.5 | 1.0 (-0.1, 2.1) | -0.6 (-2.2, 1.1) | 0.520 |
| HbA1c (%) | 0 | 5.7 |  | 5.5 |  |  |  |
|  | 12 | 5.7 | 0.0 (-0.1, 0.0) | 5.5 | 0.0 (-0.1, 0.0) | -0.0 (-0.1, 0.1) | 0.763 |
| Glucose (mg/dL) | 0 | 100.6 |  | 96.2 |  |  |  |
|  | 12 | 95.9 | -4.7 (-8.5, -0.8) | 91.2 | -4.9 (-7.6, -2.2) | 1.0 (-3.6, 5.5) | 0.678 |
| Insulin (mg/dL) | 0 | 10.5 |  | 9.2 |  |  |  |
|  | 12 | 8.8 | -1.0 (-3.3, 1.3) | 9.8 | 0.7 (-0.6, 2.0) | -1.3 (-3.3, 0.7) | 0.203 |
| HOMA-B index | 0 | 104.1 |  | 109.1 |  |  |  |
|  | 12 | 136.3 | 37.9 (-25.1, 100.9) | 134.6 | 26.4 (6.1, 46.8) | 7.0 (-54.7, 68.7) | 0.822 |
| TyG index | 0 | 8.5 |  | 8.4 |  |  |  |
|  | 12 | 8.3 | -0.2 (-0.3, -0.1) | 8.2 | -0.2 (-0.3, -0.1) | -0.0 (-0.14, 0.1) | 0.894 |
| Homocysteine (umol/L) | 0 | 12.0 |  | 12.1 |  |  |  |
|  | 12 | 12.3 | 0.3 (-0.2, 0.8) | 11.5 | -0.5 (-0.9, -0.2) | 0.8 (0.2, 1.4) | **0.006** |
| Vitamin B12 (mg/dL) | 0 | 483.3 |  | 478.1 |  |  |  |
|  | 12 | 427.9 | -44.1 (-79.3, -8.9) | 425.1 | -59.4 (-114.3, -4.5) | 9.4 (-40.4, 59.3) | 0.708 |
| TSH (mg/dL) | 0 | 2.0 |  | 2.2 |  |  |  |
|  | 12 | 1.9 | -0.1 (-0.3, 0.0) | 2.0 | -0.2 (-0.4, 0.0) | -0.0 (-0.2, 0.2) | 0.980 |
| Fibrinogen (mg/dL) | 0 | 351.5 |  | 354.1 |  |  |  |
|  | 12 | 434.9 | 74.6 (49.0, 100.3) | 418.4 | 63.7 (39.9, 87.5) | 10.6 (-22.6, 43.8) | 0.525 |
| EGCG= epigallocatechin-3-gallate. MLI= multimodal intervention. CAIDE= Cardiovascular Risk Factors, Aging, and Dementia index. EGCG= epigallocatechin-3-gallate. HbA1c= glycosylated hemoglobin. HDL-c= high density lipoprotein cholesterol. HOMA-B= homeostasis model assessment of beta cell function index. LDL-c= low density lipoprotein cholesterol. TSH= Thyroid-stimulating hormone. TyG= triglyceride-glucose index. *Adjusted mean differences (AMD) are obtained from ANCOVA models for differences from baseline adjusted for baseline scores. In the case of positive changes from baseline, a positive value of the AMD indicates larger changes in the MLI+EGCG group. In the case of negative changes from baseline, a negative value of the AMD indicates larger absolute changes in the MLI+EGCG group. | | | | | | | |

### Table 6. Intervention effects on lifestyle risk factors in the modified intention-to-treat population.

|  |  | **MLI+EGCG (n=51)** | | **MLI+placebo (n=50)** | | **MLI+EGCG vs. MLI+placebo** | |
| --- | --- | --- | --- | --- | --- | --- | --- |
|  | **Month** | **Mean** | **Mean Change  (95%CI)** | **Mean** | **Mean Change (95%CI)** | **Adjusted mean difference***  **of changes from month 0 (95%CI)** | **p-value** |
| MedDiet adherence (MEDAS-14) | 0 | 7.8 |  | 8.0 |  |  |  |
|  | 12 | 11.4 | 3.5 (3.0, 4.1) | 11.0 | 3.0 (2.4, 3.5) | 0.5 (-0.1, 1.1) | 0.081 |
|  | 15 | 11.6 | 3.6 (3.1, 4.2) | 10.8 | 2.8 (2.2, 3.4) | 0.8 (0.1, 1.4) | **0.017** |
| Physical activity (MET-min/week) | 0 | 3528 |  | 3197.4 |  |  |  |
|  | 12 | 4240 | 466 (-163, 1096) | 4111.4 | 867 (225, 1509) | -209 (-1049, 632) | 0.623 |
|  | 15 | 4549 | 774 (-415, 1963) | 3563.8 | 371 (-242, 984) | 650 (-612, 1911) | 0.309 |
| Grip strength, dominant hand (kg) | 0 | 30.0 |  | 30.6 |  |  |  |
|  | 12 | 30.4 | -0.4 (-1.2, 0.4) | 30.1 | -0.8 (-1.7, 0.1) | 0.4 (-0.8, 1.5) | 0.546 |
|  | 15 | 30.6 | -0.2 (-1.2, 0.8) | 30.1 | -0.8 (-1.7, 0.2) | 0.6 (-0.8, 1.9) | 0.407 |
| 5-Times sit-to-stand test (seconds) | 0 | 10.5 |  | 11.5 |  |  |  |
|  | 12 | 8.8 | -1.2 (-2.1, -0.3) | 9.2 | -2.3 (-3.3, -1.3) | 0.1 (-0.7, 1.0) | 0.785 |
|  | 15 | 8.3 | -1.7 (-2.6, -0.7) | 8.7 | -2.7 (-3.6, -1.8) | 0.1 (-0.8, 0.9) | 0.881 |
| Sleep quality (PSQI) | 0 | 6.3 |  | 6.7 |  |  |  |
|  | 12 | 6.4 | -0.1 (-1.0, 0.8) | 6.3 | -0.4 (-1.3, 0.5) | 0.3 (-0.7, 1.3) | 0.600 |
|  | 15 | 6.2 | -0.3 (-1.2, 0.6) | 6.4 | -0.6 (-1.5, 0.3) | 0.1 (-0.9, 1.2) | 0.819 |
| Excessive daytime sleepiness (ESS) | 0 | 6.6 |  | 6.7 |  |  |  |
|  | 12 | 6.5 | -0.4 (-1.2, 0.4) | 5.9 | -0.8 (-1.8, 0.2) | 0.5 (-0.6, 1.7) | 0.333 |
|  | 15 | 6.2 | -0.5 (-1.5, 0.4) | 6.2 | -0.6 (-1.4, 0.3) | 0.0 (-1.2, 1.2) | 0.960 |
| Olfactory functioning (UPSIT) | 0 | 28.5 |  | 28.5 |  |  |  |
|  | 12 | 27.9 | -0.9 (-2.0, 0.2) | 27.5 | -0.9 (-2.1, 0.3) | 0.1 (-1.3, 1.5) | 0.853 |
| EGCG= epigallocatechin-3-gallate. MLI= multimodal intervention. ESS= Epworth sleepiness scale. MEDAS-14= 14-point Mediterranean Diet Adherence Screener. METs= metabolic equivalent tasks. PSQI= Pittsburg Sleep Quality Index. UPSIT= University of Pennsylvania Smell Identification Test. Adjusted mean differences (AMD) are obtained from ANCOVA models for differences from baseline adjusted for baseline scores. In the case of positive changes from baseline, a positive value of the AMD indicates larger changes in the MLI+EGCG group. In the case of negative changes from baseline, a negative value of the AMD indicates larger absolute changes in the MLI+EGCG group. | | | | | | | |

### Table 7. Intervention effects on functional fitness evaluated with the Senior Fitness Test battery in the modified intention-to-treat population.

|  |  | **MLI+EGCG (n=51)** | | **MLI+placebo (n=50)** | | **MLI+EGCG vs MLI+placebo** | |
| --- | --- | --- | --- | --- | --- | --- | --- |
|  | **Month** | **Mean** | **Mean Change  (95%CI)** | **Mean** | **Mean Change (95%CI)** | **Adjusted mean difference***  **of changes from month 0 (95%CI)** | **p-value** |
| 30-Second Arm Curl Test (rep.)  (*upper-body muscle strength*) | 0 | 20.0 |  | 20.4 |  |  |  |
|  | 12 | 24.1 | 4.1 (2.6, 5.5) | 25.8 | 5.1 (3.8, 6.3) | -1.2 (-3.0, 0.6) | 0.190 |
| 30-Second Chair Stand Test (rep.) (*lower-body muscle strength*) | 0 | 18.1 |  | 17.5 |  |  |  |
|  | 12 | 23.9 | 5.7 (3.7, 7.7) | 23.9 | 6.1 (4.4, 7.8) | -0.3 (-2.8, 2.3) | 0.836 |
| 8-Foot Up-and-Go Test (seconds) (*agility and dynamic balance*) | 0 | 4.2 |  | 3.9 |  |  |  |
|  | 12 | 3.6 | -0.3 (-0.6, 0.0) | 3.6 | -0.3 (-0.5, -0.1) | 0.1 (-0.2, 0.3) | 0.750 |
| 6-Minute Walk Test (meters) (*aerobic endurance*) | 0 | 573.7 |  | 589.5 |  |  |  |
|  | 12 | 646.3 | 52.5 (38.2, 66.8) | 646.1 | 56.9 (44.6, 69.2) | -3.9 (-22.5, 14.7) | 0.677 |
| 2-Minute Step Test (rep.) (*aerobic endurance*) | 0 | 82.0 |  | 80.5 |  |  |  |
|  | 12 | 97.4 | 16.4 (11.0, 21.8) | 98.6 | 16.4 (10.8, 22.1) | -1.2 (-8.1, 5.7) | 0.732 |
| Back Scratch Test (cm) (*upper-body flexibility*) | 0 | 3.4 |  | 0.2 |  |  |  |
|  | 12 | 4.1 | 0.5 (-0.5, 1.5) | 0.3 | 1.1 (0.0, 2.2) | -0.1 (-1.6, 1.4) | 0.934 |
| Chair Sit-and-Reach Test (cm) (*lower-body flexibility*) | 0 | 7.9 |  | 5.3 |  |  |  |
|  | 12 | 9.3 | 2.6 (0.3, 4.8) | 6.0 | -0.1 (-2.5, 2.2) | 2.9 (-0.3, 6.2) | 0.075 |
| EGCG= epigallocatechin-3-gallate. MLI= multimodal intervention. Rep= number of repetitions. The Senior Fitness Test battery was available in a sample of 81 participants at baseline, 72 participants at 6 months, and 72 participants at 12 months. Adjusted mean differences (AMD) are obtained from ANCOVA models for differences from baseline adjusted for baseline scores. In the case of positive changes from baseline, a positive value of the AMD indicates larger changes in the MLI+EGCG group. In the case of negative changes from baseline, a negative value of the AMD indicates larger absolute changes in the MLI+EGCG group | | | | | | | |

### Table 8. Intervention effects on quality of life, mental health, and adaptive behavior in the modified intention-to-treat population.

|  |  | **MLI+EGCG (n=51)** | | **MLI+placebo (n=50)** | | **MLI+EGCG vs MLI+placebo** |  |
| --- | --- | --- | --- | --- | --- | --- | --- |
|  | **Month** | **Mean** | **Mean Change  (95%CI)** | **Mean** | **Mean Change (95%CI)** | **Adjusted mean difference***  **of changes from month 0 (95%CI)** | **p-value** |
| WHOQOL-BREF  Environmental domain | 0 | 72.5 |  | 75.6 |  |  |  |
|  | 12 | 78.9 | 5.4 (1.8, 9.0) | 75.9 | -0.1 (-2.6, 2.3) | 4.8 (0.8, 8.8) | **0.020** |
|  | 15 | 75.1 | 1.9 (-1.5, 5.4) | 74.8 | -0.8 (-3.9, 2.3) | 2.1 (-2.3, 6.6) | 0.335 |
| WHOQOL-BREF  Social domain | 0 | 60.3 |  | 59.8 |  |  |  |
|  | 12 | 67.1 | 6.2 (-0.9, 13.2) | 62.4 | 1.0 (-3.0, 5.0) | 4.9 (-1.8, 11.5) | 0.149 |
|  | 15 | 63.5 | 3.7 (-2.6, 10.0) | 60.2 | -0.5 (-4.8, 3.8) | 3.7 (-2.5, 9.8) | 0.238 |
| WHOQOL-BREF  Psychological domain | 0 | 67.6 |  | 69.2 |  |  |  |
|  | 12 | 73.8 | 5.2 (1.6, 8.7) | 73.1 | 2.5 (-0.4, 5.4) | 2.4 (-2.0, 6.9) | 0.281 |
|  | 15 | 70.7 | 2.6 (-0.5, 5.7) | 72.3 | 2.5 (-0.5, 5.6) | -0.2 (-4.4, 4.1) | 0.935 |
| WHOQOL-BREF  Physical domain | 0 | 74.0 |  | 75.8 |  |  |  |
|  | 12 | 77.4 | 2.7 (-2.5, 7.9) | 76.6 | -0.0 (-3.3, 3.3) | 1.8 (-3.6, 7.3) | 0.508 |
|  | 15 | 76.8 | 2.5 (-1.7, 6.7) | 78.3 | 2.5 (-0.9, 5.8) | -0.7 (-5.4, 4.0) | 0.769 |
| WHOQOL-BREF  Overall health | 0 | 3.8 |  | 3.5 |  |  |  |
|  | 12 | 3.8 | 0.0 (-0.2, 0.3) | 3.6 | 0.1 (-0.2, 0.4) | 0.0 (-0.3, 0.4) | 0.917 |
|  | 15 | 3.9 | 0.1 (-0.1, 0.4) | 3.7 | 0.2 (-0.1, 0.4) | 0.1 (-0.2, 0.4) | 0.622 |
| WHOQOL-BREF  Overall quality of life | 0 | 4.0 |  | 3.9 |  |  |  |
|  | 12 | 4.2 | 0.2 (-0.1, 0.5) | 4.0 | 0.1 (-0.1, 0.3) | 0.1 (-0.2, 0.4) | 0.397 |
|  | 15 | 4.0 | 0.0 (-0.2, 0.3) | 4.0 | 0.1 (-0.1, 0.3) | -0.0 (-0.3, 0.3) | 0.956 |
| EQ-5D-5L index | 0 | 0.9 |  | 0.9 |  |  |  |
|  | 12 | 0.9 | 0.0 (0.0, 0.0) | 0.9 | 0.0 (0.0, 0.0) | -0.0 (-0.1, 0.1) | 0.798 |
|  | 15 | 0.9 | 0.0 (0.0, 0.0) | 0.9 | 0.0 (0.0, 0.0) | 0.0 (-0.1, 0.1) | 0.489 |
| EQ-5D-5L VAS | 0 | 78.8 |  | 75.8 |  |  |  |
|  | 12 | 79.1 | -1.3 (-6.5, 3.9) | 79.7 | 3.9 (-0.8, 8.6) | -2.1 (-8.0, 3.9) | 0.489 |
|  | 15 | 82.3 | 1.6 (-2.1, 5.2) | 81.2 | 5.2 (1.5, 9.0) | -0.9 (-5.3, 3.4) | 0.672 |
| GHQ-28 total score | 0 | 3.2 |  | 2.9 |  |  |  |
|  | 12 | 2.8 | -0.5 (-1.2, 0.2) | 2.7 | -0.3 (-1.1, 0.4) | -0.1 (-1.0, 0.9) | 0.876 |
|  | 15 | 2.9 | -0.4 (-1.1, 0.3) | 2.7 | -0.3 (-1.3, 0.8) | -0.0 (-1.2, 1.2) | 0.996 |
| HADS total score | 0 | 5.5 |  | 6.5 |  |  |  |
|  | 12 | 6.4 | 0.9 (-0.8, 2.6) | 7.0 | 0.4 (-0.9, 1.7) | 0.2 (-1.9, 2.3) | 0.821 |
|  | 15 | 5.2 | -0.3 (-2.2, 1.5) | 5.9 | -0.7 (-2.3, 0.9) | -0.2 (-2.5, 2.1) | 0.886 |
| ABAS-II Total score | 0 | 101.8 |  | 99.6 |  |  |  |
|  | 12 | 100.6 | -0.2 (-4.9, 4.6) | 100.3 | 1.2 (-6.3, 8.8) | -1.0 (-9.2, 7.2) | 0.806 |
|  | 15 | 103.0 | 2.0 (-2.3, 6.3) | 103.8 | 4.1 (0.3, 8.0) | -1.7 (-6.8, 3.4) | 0.499 |
| EGCG= epigallocatechin-3-gallate. MLI= multimodal intervention. EQ-5D-5L= Spanish version of the EuroQol 5 dimensions 5 Levels. GHQ-28= General Health Questionnaire. HADS= Hospital Anxiety and Depression Scale. VAS= visual analogue scale. WHOQOL-BREF= Spanish version of the World Health Organization quality of life questionnaire. Adjusted mean differences (AMD) are obtained from ANCOVA models for differences from baseline adjusted for baseline scores. In the case of positive changes from baseline, a positive value of the AMD indicates larger changes in the MLI+EGCG group. In the case of negative changes from baseline, a negative value of the AMD indicates larger absolute changes in the MLI+EGCG group. | | | | | | | |

### Table 9. Adherence to MLI components in the modified intention-to-treat population.

|  |  |  |  | **Group differences** | | | **Sex differences** | | |
| --- | --- | --- | --- | --- | --- | --- | --- | --- | --- |
|  |  |  | **All MLI** | **MLI+EGCG** | **MLI+placebo** | **P-value*** | **Women** | **Men** | **P-value*** |
|  |  |  | **(N=101)** | **(N=51)** | **(N=50)** |  | **(N=67)** | **(N=34)** |  |
| **Individual Nutritional counselling visits (9 sessions)** | Average participation (%) | *Mean (SD)* | 89.9 (30.1) | 88.8 (31.6) | 91.1 (28.6) | 0.267 | 89.1 (31.2) | 91.5 (27.9) | 0.261 |
|  | Distribution | *N (%)* |  |  |  |  |  |  |  |
|  | ≤6 sessions |  | 6 (5.9) | 4 (7.8) | 2 (4.0) | 0.225 | 6 (9.0) | 0 (0.0) | 0.155 |
|  | 7 sessions |  | 18 (17.8) | 11 (21.6) | 7 (14.0) |  | 14 (20.9) | 4 (11.8) |  |
|  | 8 sessions |  | 44 (43.6) | 24 (47.1) | 20 (40.0) |  | 26 (38.8) | 18 (52.9) |  |
|  | 9 sessions |  | 33 (32.7) | 12 (23.5) | 21 (42.0) |  | 21 (31.3) | 12 (35.3) |  |
| **Structured Physical Activity sessions in the gymnasium  (1/week)** | Average participation (%) | *Mean (SD)* | 62.4 (38.0) | 60.2 (38.7) | 64.6 (37.2) | 0.057 | 62.7 (37.8) | 61.9 (38.3) | 0.718 |
|  | Distribution | *N (%)* |  |  |  |  |  |  |  |
|  | <25% of participation |  | 17 (16.8) | 8 (15.7) | 9 (18.0) | 0.517 | 9 (13.4) | 8 (23.5) | 0.054 |
|  | 25-50% of participation |  | 12 (11.9) | 8 (15.7) | 4 (8.0) |  | 12 (17.9) | 0 (0.0) |  |
|  | 50-75% of participation |  | 25 (24.8) | 14 (27.5) | 11 (22.0) |  | 16 (23.9) | 9 (26.5) |  |
|  | ≥75% of participation |  | 47 (46.5) | 21 (41.2) | 26 (52.0) |  | 30 (44.8) | 17 (50.0) |  |
| **Cognitive training (3 /week)** | Average monthly participation (%) |  | 72.0 (31.1) | 72.3 (31.3) | 71.8 (31.0) | 0.798 | 73.1 (30.2) | 70.0 (32.8) | 0.094 |
|  | Distribution |  | 11 (10.9) | 7 (13.7) | 4 (8.0) | 0.658 | 7 (10.4) | 4 (11.8) | 0.703 |
|  | <25% of sessions |  | 10 (9.9) | 5 (9.8) | 5 (10.0) |  | 8 (11.9) | 2 (5.9) |  |
|  | 25-50% of sessions |  | 22 (21.8) | 9 (17.6) | 13 (26.0) |  | 13 (19.4) | 9 (26.5) |  |
|  | 50-75% of sessions |  | 58 (57.4) | 30 (58.8) | 28 (56.0) |  | 39 (58.2) | 19 (55.9) |  |
|  | ≥75% of sessions |  | 11 (10.9) | 7 (13.7) | 4 (8.0) | 0.658 | 7 (10.4) | 4 (11.8) | 0.703 |
| **Psychoeducation sessions (10 sessions)** | Average participation (%) | *Mean (SD)* | 79.1 (40.7) | 77.8 (41.6) | 80.4 (39.8) | 0.320 | 81.6 (38.8) | 74.4 (43.7) | **0.009** |
|  | Distribution | *N (%)* |  |  |  |  |  |  |  |
|  | 0-3 sessions |  | 8 (7.9) | 5 (9.8) | 3 (6.0) | 0.336 | 6 (9.0) | 2 (5.9) | 0.56 |
|  | 4-6 sessions |  | 21 (20.8) | 13 (25.5) | 8 (16.0) |  | 12 (17.9) | 9 (26.5) |  |
|  | 7-10 sessions |  | 72 (71.3) | 33 (64.7) | 39 (78.0) |  | 49 (73.1) | 23 (67.6) |  |
| **EMAs (daily)** | Average weekly compliance (%) | *Mean (SD)* | 88.9 (17.5) | 87.9 (18.2) | 89.9 (16.7) | **<0.001** | 88.5 (17.9) | 89.7 (16.5) | **0.017** |
|  | Distribution | *N (%)* |  |  |  |  |  |  |  |
|  | 50-75% of compliance |  | 6 (5.9) | 4 (7.8) | 2 (4.0) | 0.692 | 4 (6.0) | 2 (5.9) | 0.999 |
|  | ≥75% of compliance |  | 95 (94.1) | 47 (92.2) | 48 (96.0) |  | 63 (94.0) | 32 (94.1) |  |
| **Fitbit (daily)** | Average monthly valid data (%) | *Mean (SD)* | 83.9 (36.8) | 82.4 (38.1) | 85.3 (35.4) | **<0.001** | 81.5 (38.8) | 88.4 (32.0) | **<0.001** |
|  | Distribution | *N (%)* |  |  |  |  |  |  |  |
|  | 25-50% of days |  | 7 (6.9) | 5 (9.8) | 2 (4.0) | 0.553 | 6 (9.0) | 1 (2.9) | 0.324 |
|  | 50-75% of days |  | 15 (14.9) | 6 (11.8) | 9 (18.0) |  | 11 (16.4) | 4 (11.8) |  |
|  | 75-90% of days |  | 20 (19.8) | 11 (21.6) | 9 (18.0) |  | 15 (22.4) | 5 (14.7) |  |
|  | ≥90% of days |  | 59 (58.4) | 29 (56.9) | 30 (60.0) |  | 35 (52.2) | 24 (70.6) |  |
| EGCG= epigallocatchin-3-gallate. MLI= multimodal intervention. EMAs= ecological momentary assessments. | | | | | | | | | |
| *The Chi-square test was used to assess differences between groups in adherence categories, and the t-test was used to compare the average adherence (continuous) in each domain between MLI groups | | | | | | | | | |

### Table 10. Determination of EGCG in plasma as an indicator of treatment compliance in the modified intention-to-treat population.

|  |  | **Participants with available information** | **EGCG positive (≥10 ng/mL)** |
| --- | --- | --- | --- |
| **Time point** | **Group** |  | **n (%)** |
| 6 months | NRG (n=25) | 21 | 0 (0%) |
|  | MLI+EGCG (n=51) | 50 | 45 (90.0%) |
|  | MLI+Placebo (n=50) | 50 | 0 (0%) |
| 12 months | NRG (n=25) | 25 | 1 (4%) |
|  | MLI+EGCG (n=47) | 47 | 40 (85.1%) |
|  | MLI+Placebo (n=49) | 48 | 1 (2.1%) |
| NRG= non-randomized. EGCG= epigallocatchin-3-gallate. MLI= multimodal intervention. | | | |

# Supplementary Figures

###
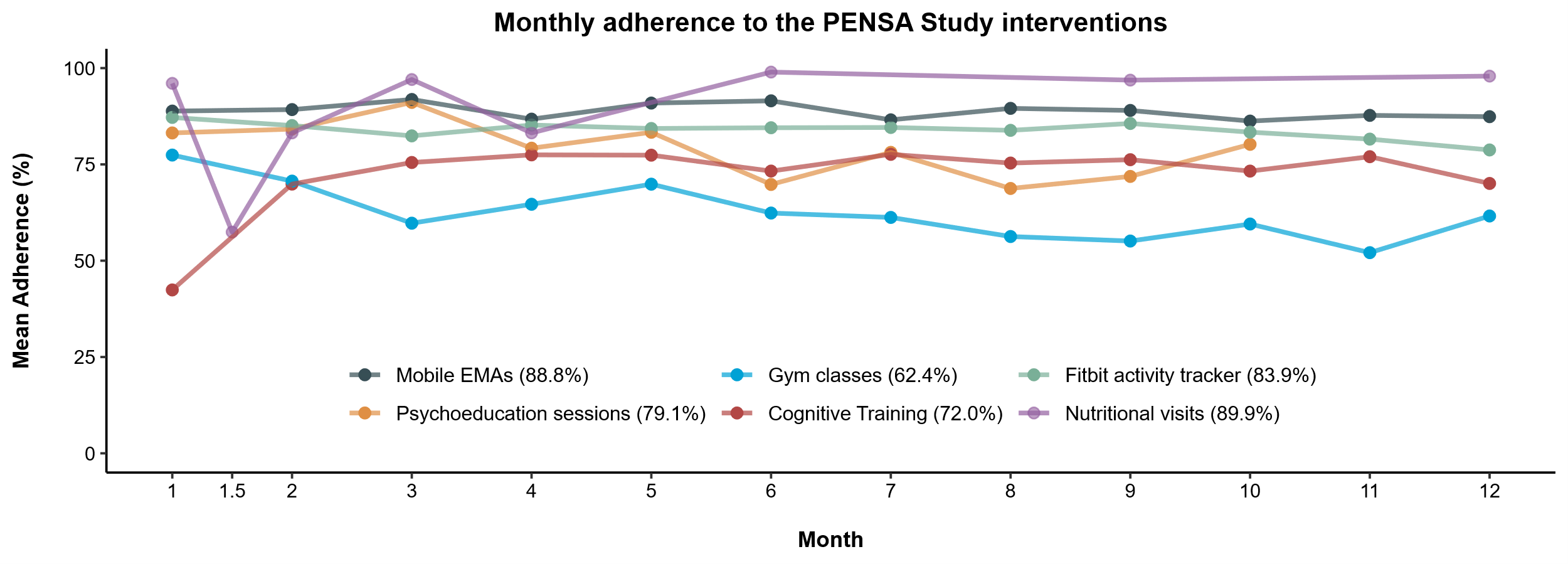
Figure 1. Monthly adherence to the multimodal intervention components.

EMAs= Ecological Momentary Assessments.

# The PENSA Study Working Group

| Aldea-Perona, Ana | Hospital del Mar Research Institute, Barcelona, Spain | [aaldea@researchmar.net](mailto:aaldea@researchmar.net) |
| --- | --- | --- |
| Boronat, Anna | Hospital del Mar Research Institute, Barcelona, Spain | [aboronat@researchmar.net](mailto:aboronat@researchmar.net) |
| Cuenca-Royo, Aida | Hospital del Mar Research Institute, Barcelona, Spain | [acuenca@researchmar.net](mailto:acuenca@researchmar.net) |
| de la Torre, Rafael | Hospital del Mar Research Institute, Barcelona, Spain | [rtorre2@researchmar.net](mailto:rtorre2@researchmar.net) |
| Diaz-Pellicer, Patricia | Hospital del Mar Research Institute, Barcelona, Spain | [pdiaz@psmar.cat](mailto:pdiaz@psmar.cat) |
| Dierssen, Mara | Centre for Genomic Regulation, Barcelona, Spain | [mara.dierssen@crg.eu](mailto:mara.dierssen@crg.eu) |
| Domingo-Gispert, Juan | Barcelonaβeta Brain Research Center, Barcelona, Spain | [jdgispert@barcelonabeta.org](mailto:jdgispert@barcelonabeta.org) |
| Fauria, Karine | Barcelonaβeta Brain Research Center, Barcelona, Spain | [kfauria@barcelonabeta.org](mailto:kfauria@barcelonabeta.org) |
| Forcano, Laura | Hospital del Mar Research Institute, Barcelona, Spain | [lforcano@researchmar.net](mailto:lforcano@researchmar.net) |
| Fusté, David | Barcelonaβeta Brain Research Center, Barcelona, Spain | [dfuste@barcelonabeta.org](mailto:dfuste@barcelonabeta.org) |
| Gomis-González, Maria | Hospital del Mar Research Institute, Barcelona, Spain | [mgomis@researchmar.net](mailto:mgomis@researchmar.net) |
| González de Echevarri, José M | Barcelonaβeta Brain Research Center, Barcelona, Spain | [jgonzalez@barcelonabeta.org](mailto:jgonzalez@barcelonabeta.org) |
| González Tartière, Pilar | Barcelonaβeta Brain Research Center, Barcelona, Spain | [pgonzalez@barcelonabeta.org](mailto:pgonzalez@barcelonabeta.org) |
| Grau-Rivera, Oriol | Barcelonaβeta Brain Research Center, Barcelona, Spain | [ograu@barcelonabeta.org](mailto:ograu@barcelonabeta.org) |
| Langohr, Klaus | Hospital del Mar Research Institute, Barcelona, Spain | [klangohr@researchmar.net](mailto:klangohr@researchmar.net) |
| Lorenzo, Thais | Hospital del Mar Research Institute, Barcelona, Spain | [tlorenzo@researchmar.net](mailto:tlorenzo@researchmar.net) |
| Mateus, Julián | Hospital del Mar Research Institute, Barcelona, Spain | [jmateus@colisee.es](mailto:jmateus@colisee.es) |
| Meneces-Cabral, Sofia | Barcelonaβeta Brain Research Center, Barcelona, Spain | [smeneces@barcelonabeta.org](mailto:smeneces@barcelonabeta.org) |
| Minguillón, Carolina | Barcelonaβeta Brain Research Center, Barcelona, Spain | [cminguillon@barcelonabeta.org](mailto:cminguillon@barcelonabeta.org) |
| Molinuevo, José Luis | H. Lundbeck A/S, København, Denmark | [jlmolinuevo@barcelonabeta.org](mailto:jlmolinuevo@barcelonabeta.org) |
| Mur-Gimeno, Esther | Escola Superior de Ciències de la Salut Tecnocampus, Universitat Pompeu Fabra, Mataró, Spain | [emur@tecnocampus.cat](mailto:emur@tecnocampus.cat) |
| Pérez-Otero, Marta | Hospital del Mar Research Institute, Barcelona, Spain | [mperez2@researchmar.net](mailto:mperez2@researchmar.net) |
| Piera, Iris | Hospital del Mar Research Institute, Barcelona, Spain | [irispiera15@gmail.com](mailto:irispiera15@gmail.com) |
| Pizarro, Nieves | Hospital del Mar Research Institute, Barcelona, Spain | [npizarro@researchmar.net](mailto:npizarro@researchmar.net) |
| Puig-Pijoan, Albert | Hospital del Mar Research Institute, Barcelona, Spain | [apuig@psmar.cat](mailto:apuig@psmar.cat) |
| Radoi, Andreea | Barcelonaβeta Brain Research Center, Barcelona, Spain | [aradoi@barcelonabeta.org](mailto:aradoi@barcelonabeta.org) |
| Sánchez-Benavides, Gonzalo | Barcelonaβeta Brain Research Center, Barcelona, Spain | [gsanchezb@barcelonabeta.org](mailto:gsanchezb@barcelonabeta.org) |
| Soldevila-Domenech, Natalia | Barcelonaβeta Brain Research Center, Barcelona, Spain | [nsoldevila@barcelonabeta.org](mailto:nsoldevila@barcelonabeta.org) |
| Soteras, Anna | Barcelonaβeta Brain Research Center, Barcelona, Spain | [asoteras@barcelonabeta.org](mailto:asoteras@barcelonabeta.org) |
| Suárez-Calvet, Marc | Barcelonaβeta Brain Research Center, Barcelona, Spain | [msuarez@barcelonabeta.org](mailto:msuarez@barcelonabeta.org) |
